# Dynamic interaction of the *Yersinia pseudotuberculosis* type three secretion system proteins LcrV and LcrG

**DOI:** 10.1002/pro.70400

**Published:** 2025-12-22

**Authors:** Jagadish Chandra Kumar Mangu, Per Rogne, Jonna Mattsson, Lucas Hultgren, Kumar D. Gahlot, Anaïs Lamy, Ronnie P.‐A. Berntsson, Lennart B.‐Å. Johansson, Matthew S. Francis, Magnus Wolf‐Watz

**Affiliations:** ^1^ Department of Chemistry Umeå University Umeå Sweden; ^2^ Department of Molecular Biology Umeå University Umeå Sweden; ^3^ Umeå Centre for Microbial Research Umeå University Umeå Sweden; ^4^ BioMS, Department of Clinical Sciences Lund University Lund Sweden; ^5^ Department of Medical Biochemistry and Biophysics Umeå University Umeå Sweden

**Keywords:** chaperone LcrG, needle tip complex, pentameric LcrV, protein conformational switch, type III secretion system, *Yersinia* pathogenicity

## Abstract

*Yersinia* pathogenicity is dependent on polarized translocation of effector proteins via the type III secretion system (T3SS). The tip complex situated on the needle structure of the T3SS is required for contact with the eukaryotic host membrane and is to an extent composed of pentameric LcrV. LcrV is a multifunctional protein that also acts as a regulator of the T3SS by virtue of forming a high‐affinity complex in the cytoplasm with its chaperone, LcrG. By employing a structure‐based approach centered on mass spectrometry, FRET and NMR spectroscopy, we demonstrated that the LcrV‐LcrG complex is best described as a multivalent complex, and that the N‐terminal domain of LcrV contributes by negatively affecting the LcrG binding affinity. The N‐terminal domain of LcrV is dynamic and undergoes a conformational change to accommodate LcrG binding. ^19^F NMR spectroscopy analysis suggests that the conformational change is an intrinsic property of the protein, which agrees with a conformational selection model. An analysis of effector secretion into a culture supernatant demonstrated that the low synthesis and low secretion phenotypes of a LcrV mutant where the N‐terminal domain has been removed are linked to the structure, interactions and stability of the LcrV N‐terminal domain. In summary, our results add insights into the dynamics of LcrV and its complex with LcrG.

## 1 INTRODUCTION

*Yersinia pseudotuberculosis* is a Gram‐negative enteric pathogen that replicates in the lymphoid tissues of the infected hosts. Upon entering into the mammalian host through the oral route enteropathogenic *Yersinia* species utilize adhesins to invade the M cells of Peyer's patches thereby colonizing the lymph tissues (Fällman et al., 2001). *Yersinia* encounters several immune cells (macrophages, B cells, and T cells) in the host infected tissues. Therefore, to establish a successful infection that involves evading the cellular immune response, *Yersinia* is reliant on the *Yersinia* secretory complex (Ysc) and the *Yersinia* outer protein (Yop) gene products. The Ysc products enable assembly into the type III secretion system (T3SS) injectisome spanning through their inner and outer membranes, whereas Yop products traverse through the injectisome and are targeted inside the eukaryotic cells to promote bacterial pathogenicity and immune evasion (Coburn et al., 2007; Cornelis & Wolf‐Watz, 1997; Lee & Schneewind, 1999; Marketon et al., 2005; Mecsas et al., 2001). This mechanism of toxin delivery is conserved across many other Gram‐negative plant, animal, insect, and human pathogens (Coburn et al., 2007; McQuade & Stock, 2018; Puhar & Sansonetti, 2014). Importantly, several clinically relevant human pathogens such as *Shigella*, *Salmonella*, *Escherichia*, and *Yersinia* species employ the T3SS in eliciting a broad spectrum of infections (Coburn et al., 2007; Gaytán et al., 2016; Pinaud et al., 2018; Sanchez‐Garrido et al., 2022; Wu et al., 2023).

Assembly and regulation of the *Yersinia* T3SS and its effector secretion is a multilayered process. Upon host contact, the increase in temperature induces transcription of T3SS genes via the LcrF regulator (Yother et al., 1986), leading to assembly of the membrane spanning injectisome. However, the effector synthesis and secretion remain tightly regulated either through post‐translational blockage imparted by YopD, LcrH, and LcrQ, or through inhibition of gene expression and secretion by YopN‐TyeA, LcrV, and LcrG (Dewoody et al., 2013). Once this blockage is removed by in vivo host cell contact or in vitro calcium depletion in laboratory media, the synthesis and secretion of Yops occur (Bergman et al., 1991; Francis et al., 2001). Disruption of genes encoding negative regulators, such as YopN‐TyeA and LcrG, induces constitutive expression of the T3SS and drives effector secretion even in the absence of host cell contact (Day et al., 2003; Hamad & Nilles, 2007). In contrast, the disruption of genes encoding positive regulators, like LcrV, causes downregulation of gene expression and secretion (Figure S1) (Ligtenberg et al., 2013; Skrzypek & Straley, 1995).

There exists a relatively large body of data on the structure of the T3SS needle complex, and in particular from *Salmonella typhimurium* (Hu et al., 2020). This includes an in situ structure of the needle complex obtained with cryo‐electron tomography (Hu et al., 2017), as well as single particle structures at atomic resolution (Hu et al., 2019). Complementing these structures is a low‐resolution structure of isolated needles from *Yersinia enterocolitica* (Mueller et al., 2005), and this structure suggests that LcrV is arranged as a pentamer at the tip of the needle. Confirming the role of LcrV at the tip there exist polyclonal and monoclonal antibodies raised against LcrV with the ability to neutralize infections of immune cells (Biryukov et al., 2023; Cowan et al., 2005; Hill et al., 1997; Hotinger & May, 2020). Recently, neutralizing monoclonal antibodies were isolated from patients with chronic *Pseudomonas aeruginosa* infections and these antibodies bound to the LcrV homologue PcrV (Simonis et al., 2023). Taken together, these studies demonstrate the importance of LcrV and PcrV for infectious diseases and establish the protein as a viable target for the development of antibacterial therapy.

The needle tip protein, LcrV, is composed of 326 amino acids, part of which forms coiled‐coil motifs in the α‐helices 7 and 11 and attains a tertiary structure that resembles a dumbbell shape (Derewenda et al., 2004). The coiled‐coil motifs observed in the needle tip complexes of the related homologs in *Pseudomonas* (PcrV), *Salmonella* (SipD), *Shigella* (IpaD) and *Escherichia* (EspA) share structural conservation, and are known to interact with the needle filament as well as in the oligomerization of the needle tip. The N‐terminus of the needle tip proteins also differs structurally by adopting an alpha hairpin structure (in *Salmonella* and *Shigella*) or a globular domain (in *Yersinia* and *Pseudomonas*). Furthermore, the requirement of a cognate chaperone for the secretion of needle tip proteins is also another difference observed in the different bacterial T3SSs. In *Escherichia*, *Pseudomonas*, and *Yersinia* the needle tip protein requires a chaperone for successful secretion and oligomerization (Pais et al., 2023). The cognate LcrV‐chaperone, LcrG, is a 95‐residue disordered protein lacking detectable tertiary structure (Chaudhury et al., 2015). LcrG is presumed to wrap around and interact with the coiled‐coil motifs in LcrV (Nilles et al., 1997). During growth in conditions nonpermissive for secretion, cytoplasmic LcrG is presumed to interact with the T3SS injectisome and inhibit its function. As the pathogen switches to growth in secretion‐permissive conditions, cytoplasmic LcrV is hypothesized to bind LcrG and thereby derepress T3SS inhibition (Figure S1) (Matson & Nilles, 2002; Skryzpek & Straley, 1993). Despite previous studies, detailed structural insights into the dynamics of the LcrV–LcrG interaction and its potential relationship to T3SS function remain limited. A comprehensive investigation focusing on the structure, dynamics, and assembly mechanism of the LcrV–LcrG complex is therefore warranted. This study primarily explores the structural and biophysical aspects of the interaction, serving as a model for its assembly within the cytoplasmic space. We do not address the unresolved question of how LcrV is translocated from the cytoplasm to the tip of the maturing needle complex, nor do we examine the role of the LcrV–LcrG interaction following T3SS activation triggered by host cell contact or low‐calcium growth conditions.

Previously, deletion mutagenesis studies emphasized a few LcrV and LcrG regions needed to establish a binary interaction and influence control of the T3SS (Matson & Nilles, 2001; Nilles et al., 1997; Nilles et al., 1998; Reina et al., 2008; Sarker et al., 1998; Skryzpek & Straley, 1993; Skrzypek & Straley, 1995). Additionally, site‐directed mutagenesis studies have highlighted several important amino acids in both LcrV and LcrG that were required for the interaction yet contributed very little to maintaining T3SS control (Lawton et al., 2002; Matson & Nilles, 2001, 2002). While foundational insights into the roles of LcrV and LcrG in regulating the T3SS have been established, a detailed molecular understanding of their interaction remains lacking. To address this gap, herein we investigated the molecular interface between LcrV and LcrG using an integrated structural, biophysical, and functional approach.

## 2 RESULTS

### 2.1 Domains of LcrV that influence LcrG interaction patterns

**FIGURE 1:**
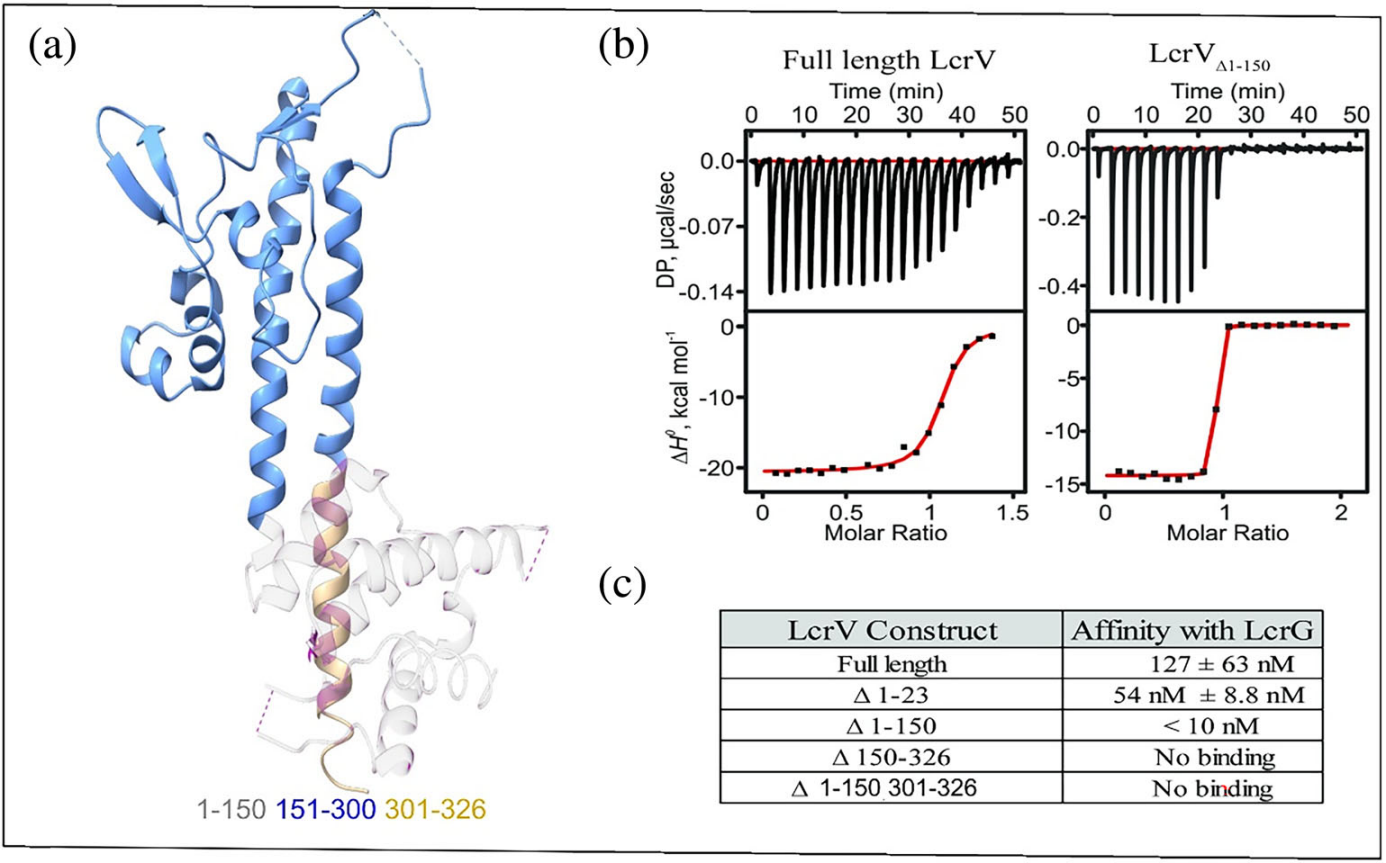
Mapping of regions in LcrV that influence the affinity towards LcrG. (a) The different regions in LcrV are highlighted on the crystal structure of LcrV (PDB ID: 1R6F; Derewenda et al., 2004). The N‐terminal domain (residues 1–150) is colored in pink and the C‐terminal coiled‐coiled α‐helix (residues 301‐326)I s colored in orange. (b) Isothermal titration calorimetry (Ligtenberg Katherine et al.) analysis of the interaction between full length LcrV and a construct lacking the N‐terminal domain (LcrV_Δ1–150_) with full length LcrG. (c) Overview of interaction affinities of LcrV constructs with full length LcrG, LcrV with a deletion of the N‐terminal domain (LcrV _Δ1–150_) LcrV with a deletion of the N‐terminal 23 residues (LcrV_Δ1–23_) displayed increased affinity towards full length LcrG whereas LcrV constructs lacking the C‐terminus abrogates interaction with LcrG. Experiments were performed at least in triplicate (*n* = 3).

Earlier studies hypothesized that LcrG could orient around and interact with the coiled‐coil domains of LcrV (Lawton et al., 2002; Matson & Nilles, 2002). To map the regions in LcrV that are important for LcrG binding, we first determined interaction affinities of full length and truncated variants of LcrV with full length LcrG using isothermal titration calorimetry (ITC). Full length LcrV bound to LcrG in a 1:1 stoichiometry with an affinity (*K*_D_) of 127 ± 63 nM (Figure 1b,c). Deletion of the N‐terminal 23 amino acid residues in LcrV resulted in a higher binding affinity relative to the full‐length protein with a *K*_D_ of 54 ± 8.8 nM. Deletion of the entire N‐terminal globular domain (i.e. residues 1–150) resulted in a binding affinity that was too strong for accurate quantification with ITC and an upper limit of the *K*_D_ was estimated at 10 nM (Figures 1b,c and S2). On the other hand, a LcrV variant with deletions in the C‐terminal alpha helix (Δ301‐326), as well as the isolated N‐terminal domain was both unable to interact with LcrG (Figure 1c). These results suggest that while the C‐terminal 26 residues of LcrV are essential for the LcrV–LcrG interaction, the N‐terminus of LcrV has a negative influence on the binding affinity since its removal results in a truncated protein with higher binding affinity toward LcrG.

### 2.2 Mapping the LcrV–LcrG interaction with masspectrometry

**FIGURE 2:**
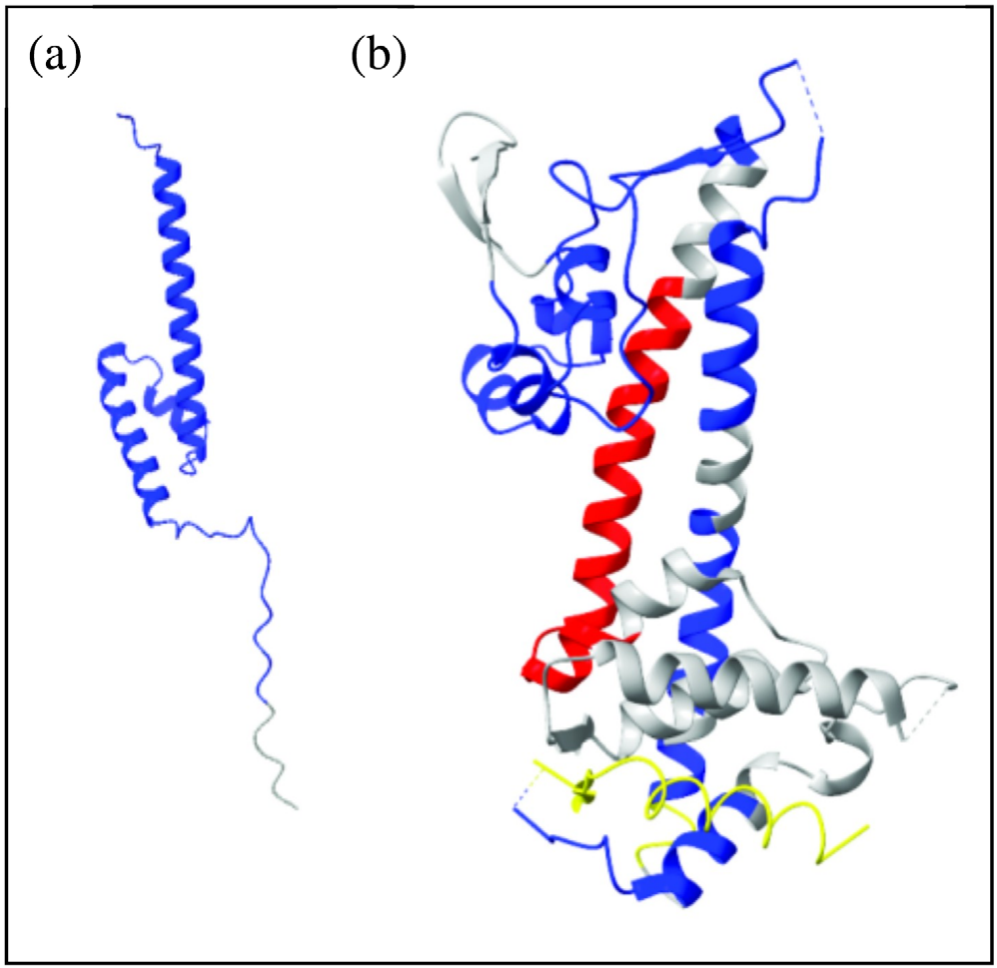
Hydrogen exchange in the LcrV‐LcrG complex from HDX‐MS. (a) AlphaFold2 model of LcrG (AFDB accession no. AF‐A0A0N9NMD5‐F1‐v4). Protein–protein interaction analysis was performed on LcrV‐LcrG by using a molar ratio of 1:1. Residues with a decreased or increased hydrogen/deuterium exchange upon LcrV and LcrG interaction are represented in blue and red in LcrV‐LcrG complex, and continuous residues with alternating rates of exchange are colored yellow. Analysis was made on triplicate samples and the statistically significant peptides (*p* < 0.01) were identified using Deuteros 2.0. (b) Changes to hydrogen/deuterium exchnage in LcrV upon complex formation is illustrated on the LcrV crystal structure (PDB ID: 1R6F; Derewenda et al., 2004).

A molecular description of the LcrV–LcrG complex requires a characterization of the interaction interface on both proteins. LcrV was shown to adopt a stable fold, while LcrG is intrinsically disordered (Chaudhury et al., 2015; Derewenda et al., 2004). Further, NMR analysis suggested that LcrG does not fold into a well‐defined structural state despite associating with LcrV in a stable complex with high affinity (*K*_D_ 127 ± 63 nM) (Figure 1b). This indicates that the interactions could be dynamic, and with some degree of disorder preserved in the protein complex (Tompa & Fuxreiter, 2008). Our extensive attempts to crystallize the LcrV–LcrG complex were unsuccessful, perhaps due to a potential dynamic nature of the LcrV and LcrG interaction. Therefore, we instead employed hydrogen to deuterium exchange mass spectrometry (HDX‐MS) to define the binding interfaces on both proteins. The basic principle of HDX experiments is to probe the likelihood that an exchangeable hydrogen, such as amide protons, is exchanged into deuterium following a rapid dilution of the protein into deuterium oxide (Hvidt & Nielsen, 1966). The rate of hydrogen to deuterium exchange is affected by hydrogen bonding and the overall stability of structural elements in the protein (Bédard et al., 2008). In proteins that undergo conformational changes upon complex formation with a partner protein, certain residues at the binding interface may exhibit a decreased exchange rate, reflecting reduced solvent accessibility or increased structural rigidity. Conversely, increased exchange rates can occur in regions where conformational rearrangements lead to enhanced solvent exposure or diminished stability. Detection of HDX with mass spectrometry (HDX‐MS) is accomplished through a rapid dilution of the protein under investigation in deuterium oxide followed by quenching of the exchange after a set time by lowering the pH of the solution. Quantification of the degree of incorporation of deuterium at exchangeable sites is then accomplished for peptide fragments following proteolytic cleavage. HDX‐MS has been applied successfully in studies of systems containing a degree of disorder and can also uncover allosteric events between proteins (Konermann & Scrosati, 2024). We quantified changes in hydrogen/deuterium exchange rates by comparing the individual profiles of free LcrV and LcrG with those observed in the LcrV–LcrG complex. The overall coverage of peptides for both proteins was significant (LcrV: 94.3%, LcrG: 99.1%, Figure S3A, C) and allowed us to portrait a comprehensive map of changes in exchange rates for both proteins. The results for LcrG are conceptually straightforward with an overall decrease in hydrogen exchange for the whole protein (residues 1–33, 19–55, 45–89). The reduction in hydrogen/deuterium exchange in LcrG upon complex formation with LcrV is illustrated on an AlphaFold2 (Varadi et al., 2024) model of the protein (Figure 2b), which had an average pDLLT score of 74.31 (AFDB accession no. AF‐A0A0N9NMD5‐F1‐v4). By interpreting the decreased exchange rates in LcrG as an increase in stability, the majority of the residues (residues 1–89 out of a total 95 residues in LcrG) appear to be stabilized upon complex formation with LcrV. This observation aligns with a previous study indicating that residues 1–73 of LcrG contain the primary interaction sites for LcrV (Chaudhury et al., 2015). The stabilization of residues 1–89 in LcrG by LcrV suggests that most of these residues, though not necessarily all, are likely to be in contact with LcrV. When the interaction is analyzed from the perspective of LcrV, continuous regions that display both decreased and increased rates of exchange were observed, rendering a scenario with a higher degree of complexity relative to that of LcrG. Peptides displaying decreased and increased exchange rates upon LcrG binding are highlighted on the LcrV structure (PDB ID: 1R6F; Derewenda et al., 2004) (Figure 2a). LcrV regions with decreased exchange rates are likely to represent the LcrG interaction interface and these regions are distributed across the protein (Figures 2a and S3B, D). There exists one continuous stretch of LcrV residues that experience increased exchange rates upon LcrG binding. These correspond to the first helix involved in the intramolecular coiled‐coil interaction. An increase in exchange can conceptually not depend on direct interaction but must depend on other changes to the protein structure. The simplest mechanism involving a structural change is a rigid‐body conformational rearrangement. A candidate segment for such a rearrangement is the N‐terminal LcrV domain that directly precedes the first coiled‐coil helix. It is therefore possible that a change in orientation of the domain will enable increased structural fluctuations of the coiled‐coil helix that are otherwise hindered by packing against the N‐terminal domain.

### 2.3 Dynamics of the LcrV–LcrG interaction

**FIGURE 3:**
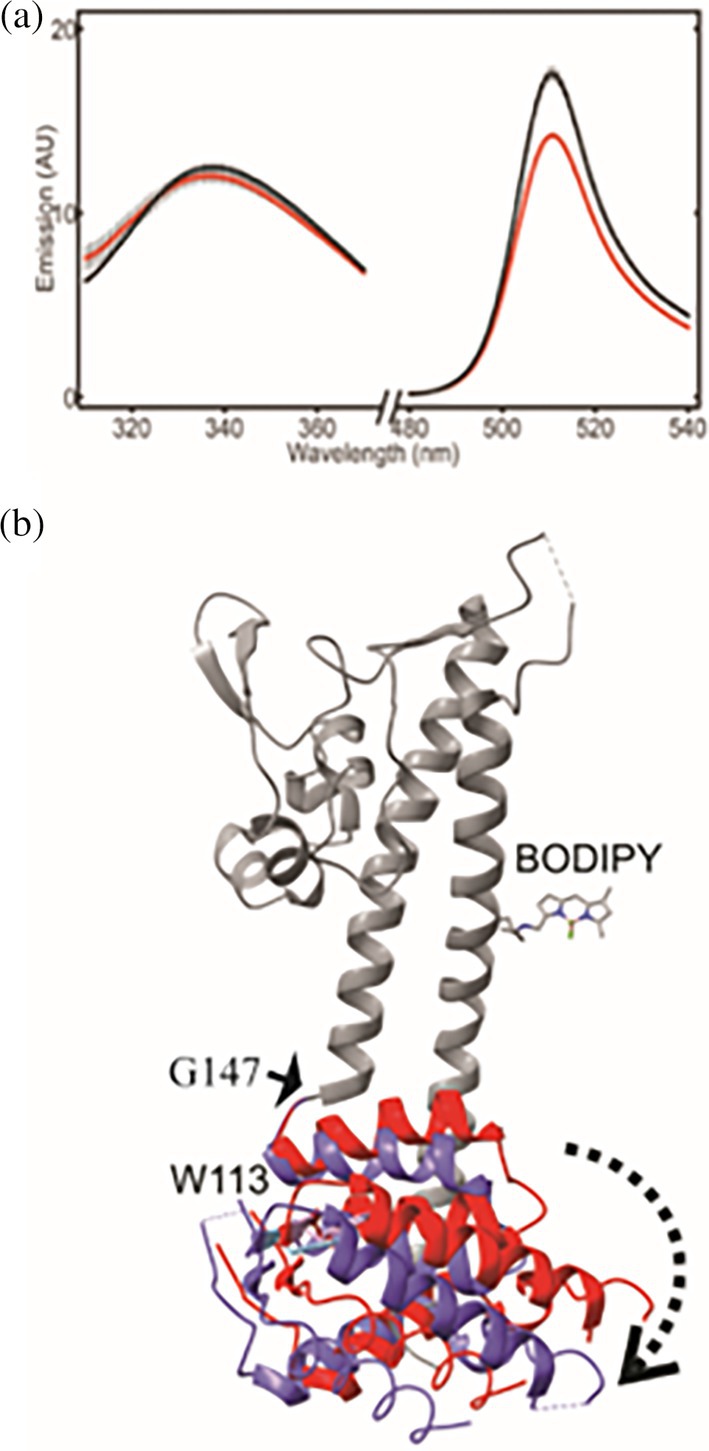
Distance measurements between the C‐terminal coiled‐coil helix and the N‐terminal domain using FRET. (a) Fluorescence emission spectra of LcrV‐BODIPY alone (black) and in complex with LcrG (red) in the Trp emission range (310–370 nm) and the BODIPY emission range (480–540 nm). In all spectra the Trp was excited at its absorption maximum of 280 nm. The data for the complex have been normalized by a factor of 1.26 due to the difference in Trp absorption caused by the absorption background of LcrG. The errors are calculated as the standard deviation of three independent measurements. (b) Crystal structure of LcrV (PDB ID: 1R6F; Derewenda et al., 2004) with the positioning of the N‐terminal from the crystal structure (red) and the position of the C‐terminal in a model where the distance between BODIPY and Trp113 have been increased by 3 Å by the interaction with LcrG and centered on rotation of the torsion angles of Gly147 (blue).

We developed our idea that the N‐terminal domain of LcrV undergoes a conformational change upon interaction with LcrG using Förster Resonance Electronic Transfer (FRET). The basic concept underlying quantification of distances with FRET measurements is the energy transfer between two chromophores, where the energy transfer scales with the inverse distance to the power of six between the chromophores. This distance dependency allows quantification of inter molecular distances in proteins by making use of equations and methodology explained in detail in Data S1. If the N‐terminal domain moves to accommodate LcrG binding, then there should be a modulation of the distance of this domain to the coiled‐coil helix of LcrV. Fortunately, LcrV has only one tryptophan residue at position 113 in the N‐terminal domain. We introduced cysteine at position Asp293 in the coiled‐coil domain and attached a fluorescent BODIPY molecule through an iodoacetamide linker. Meanwhile, the naturally occurring cysteine at position 273 was substituted for an alanine. This enabled us to validate energy transfer from the tryptophan residue to the artificial BODIPY molecule introduced into the coiled‐coil helices. The Trp/BODIPY pair is a well‐characterized FRET pair in accurately measuring molecular distances (Olofsson et al., 2006). A detailed methodology of the analysis is provided in Data S1. In short, we excited Trp113 at 280 nm and followed its changes in emissions at 339 nm and BODIPY at 511 nm in the presence and absence of LcrG (Figure 3a). Analysis of the energy transfer process revealed that the distance (center of masses) from the tryptophan to the BODIPY molecule increased from 29.8 ± 1.3 to 33 ± 1.1 Å. Hence, the FRET experiments support our hypothesis that conformational changes occur in the N‐terminal domain of LcrV upon LcrG binding. Of interest is knowing the nature of this conformational change. Since the N‐terminal domain is linked to the coiled‐coil helices through a turn element that contains a glycine residue (Gly147), it is entirely possible that the N‐terminal pivots around a change in backbone torsion angles for this glycine. In Figure 3b, we explored this prospect by modeling how a change in the glycine torsion angles translates into a conformational change, resulting in a distance increase of ~3 Å.

**FIGURE 4:**
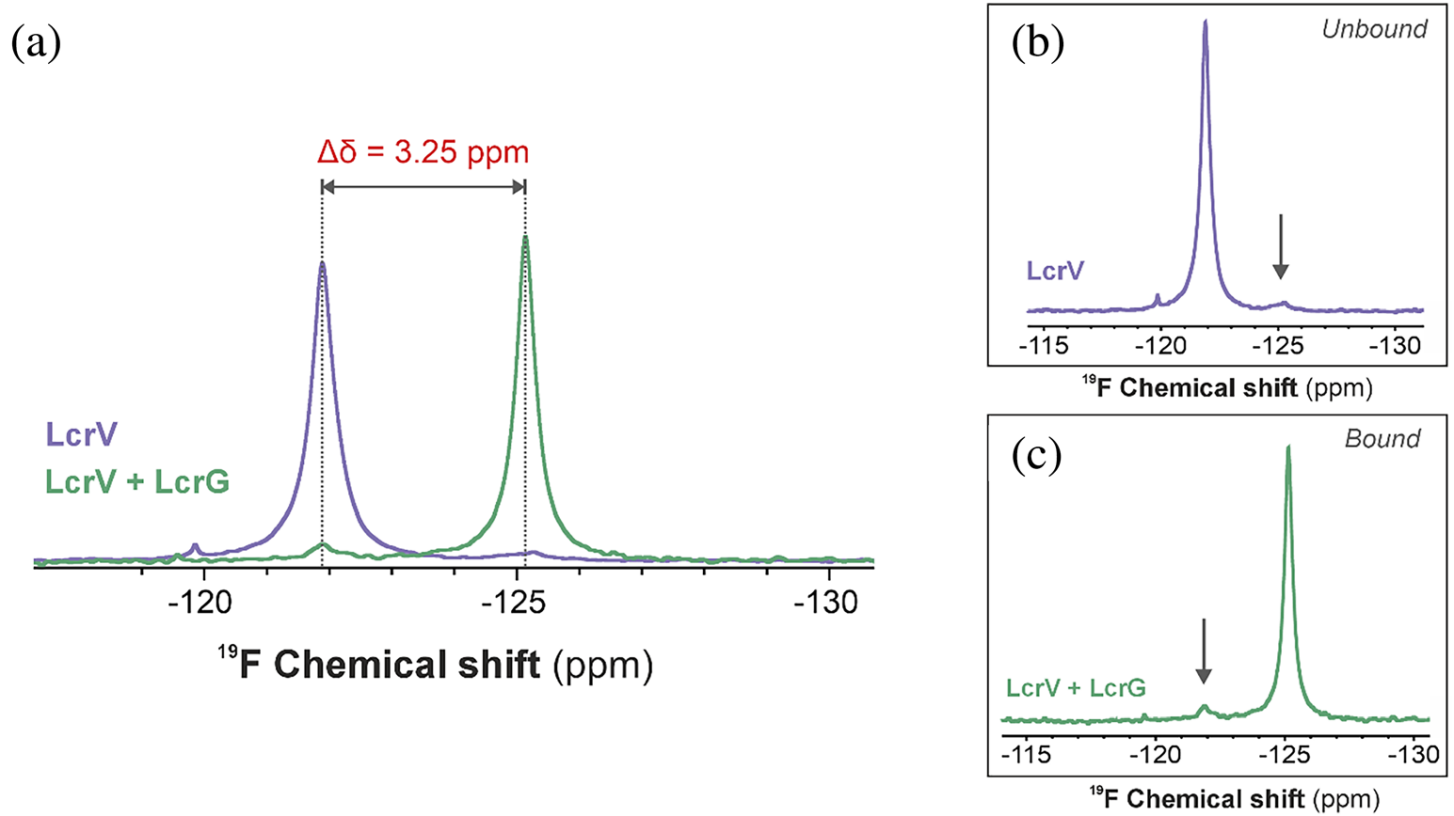
^19^F NMR spectra of 5‐fluoro‐Trp labeled LcrV in its free and LcrG bound states. (a) The 5‐fluoro‐Trp labeled LcrV displays one main peak at 121.88 ppm in its free state (purple spectrum). After addition of LcrG, the peak shifts to 125.13 ppm (green spectrum), clearly demonstrating that LcrV binds to LcrG to form a tightly bound complex. (b) LcrV probes the LcrG bound conformational state in the absence of LcrG, indicated with a gray arrow and (c) LcrV in complex with LcrG occupies an unbound, “free like” conformational state even at a two‐fold excess of LcrG. The minor, unbound state is highlighted with a gray arrow.

Having established that the N‐terminal domain of LcrV undergoes a conformational change, we also aimed to characterize its dynamic signature with NMR spectroscopy. Bio‐molecular ^19^F NMR has emerged as an attractive and alternative method for studying and characterizing proteins (Bann et al., 2002; Danielson & Falke, 1996; Verma et al., 2022). The inherent properties of the ^19^F nuclei render it extremely sensitive to its local conformational and electronic environments, making it well‐suited for studying protein target interactions, as well as drug and ligand binding (Buchholz & Pomerantz, 2021; Kitevski‐LeBlanc & Prosser, 2012). We took advantage of the singular Trp113 residue in LcrV by labeling it with 5‐Fluoroindole to establish a probe for ^19^F NMR spectroscopic studies of the complex formation between LcrV and LcrG. The acquired ^19^F NMR spectrum of 5‐Fluoroindole labeled LcrV in its free form shows one main peak at approximately 121 ppm that upon the addition of a stoichiometrically excessive amount of LcrG, shifts to around 125 ppm (Figure 4a). The chemical shift difference between the two LcrV states (free and bound) is large in comparison to the linewidths of the two states (Δδ = 3.25 ppm). This observation is evidence that LcrV can bind to LcrG to form a tight complex, consistent with the nanomolar affinity observed from ITC measurements (Figure 1b). Although the NMR spectra of LcrV are dominated by the main free and LcrG bound peaks, we consistently observed minor peaks in both the free and bound spectra (Figure 4b,c). In the free LcrV spectrum, there exists a minor peak at the resonance position of LcrV in complex with LcrG. The integral of the minor peak is 2.6% relative to the free major peak (Figure 4b). The minor peak in free LcrV is consistent with a small fraction of LcrV that adopts a structural state that resembles that of LcrG bound LcrV. Bearing in mind that the position under investigation (Trp113) is located in the N‐terminal domain, it is possible that the minor state reflects a conformation where the N‐terminal domain has changed its orientation to a state similar to that in the LcrG complex. There also exists a minor peak of LcrV in the LcrG complex that resonates at the position of free LcrV. This minor state has an integral of 3.5% (Figure 4c). The experiment was performed with a two‐fold excess of LcrG, and since the dissociation constant (*K*_D_) was determined to be 127 ± 63 nM, thereby ruling out unbound LcrV as the source of the minor peak. These minor peaks and their respective resonance positions suggest that a bound‐like structure of LcrV is sampled in the free state, while a free‐like LcrV structure is sampled in the LcrG complex. Taken together, the FRET and ^19^F NMR data uncovered that LcrV undergoes a dynamic conformational change in its N‐terminal domain upon complex formation with LcrG.

### 2.4 The N‐terminal 150 residues of LcrV blocks oligomerization in vitro

**FIGURE 5:**
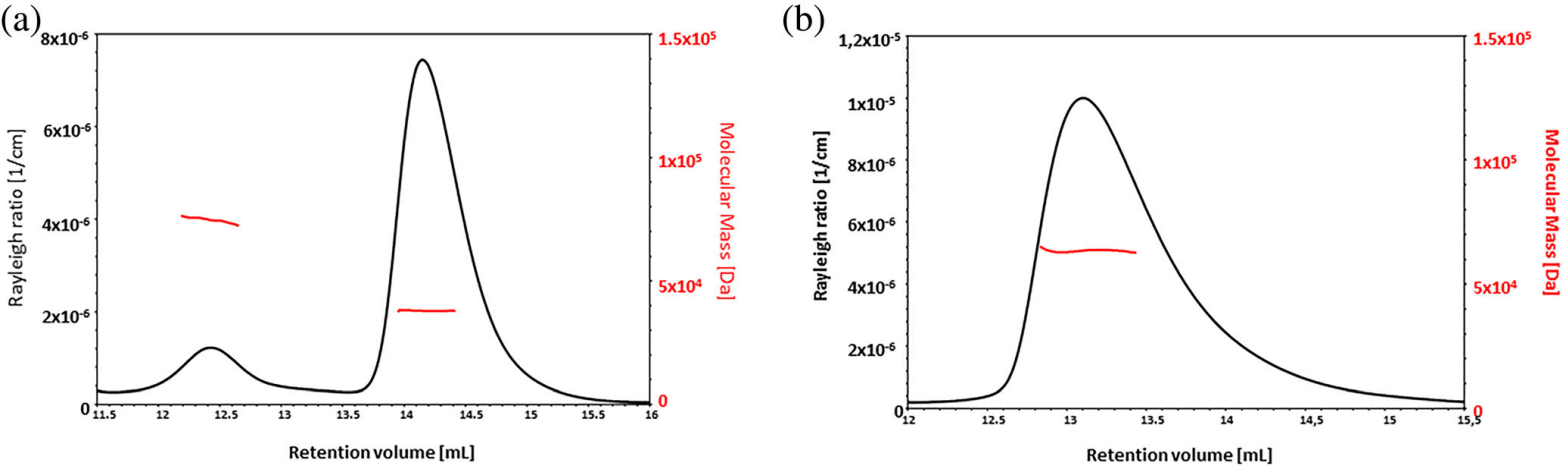
N‐terminally truncated LcrV is trimeric. SEC‐MALS elution profiles with the Rayleigh ratio and average molecular mass calculated by MALS for (a) Full length LcrV and (b) N‐terminally truncated LcrV (LcrV_Δ1–150_).

We determined the oligomeric state of LcrV using size exclusion chromatography coupled to multi‐angle light scattering (SEC‐MALS). The methodology allows an accurate analysis of molecular weights by quantification of the intensity of scattered light as the protein elutes from the size exclusion column (Some et al., 2019). Full‐length LcrV was predominantly monomeric with an estimated molecular mass (M_w_) of 38 ± 0.5 kDa, with a small fraction having a molecular mass close to that of a dimer (M_w_ 73.6 ± 0.7 kDa) (Figure 5a). Since our earlier data showed that the N‐terminus undergoes conformational changes upon LcrG binding, we also assessed the oligomeric state of the N‐terminal deletion variant (LcrV_Δ1‐150_). Surprisingly, SEC‐MALS analysis revealed that this truncated construct had a molecular weight of 63.8 ± 0.7 kDa, very close to that of a trimer (monomer has a calculated weight of 20 kDa) (Figure 5b). A monomeric aggregation state is restored when a complex with LcrG is formed (Figure S4). A similar result has been observed for the N‐terminal domain in the homologous PcrV protein from *P. aeruginosa* (Basu et al., 2014) suggesting that this behavior might be a general characteristic of bacteria harboring T3SS machinery of the Ysc‐Yop clade. The results are intriguing and reveal at least two distinct aspects of the N‐terminal domain's presence in full‐length LcrV. Firstly, its presence reduces the apparent binding affinity toward LcrG. Secondly, its removal leads to a LcrV variant that is prone to self‐assembly. Therefore, the packing of the N‐terminal domain on the C‐terminal coiled‐coil helix (Figure 1a) may mask an oligomerization site. This is pertinent given the suggestion that oligomerization of LcrV into a pentameric state is important for its function at the tip of the injectisome (Broz et al., 2007; Gébus et al., 2008; Mueller et al., 2005). It is then possible that the pentameric LcrV structure at the injectisome tip has the N‐terminal domain in a conformation that has exposed an oligomerization site.

### 2.5 Functional analysis of the LcrV N‐terminal domain

**FIGURE 6:**
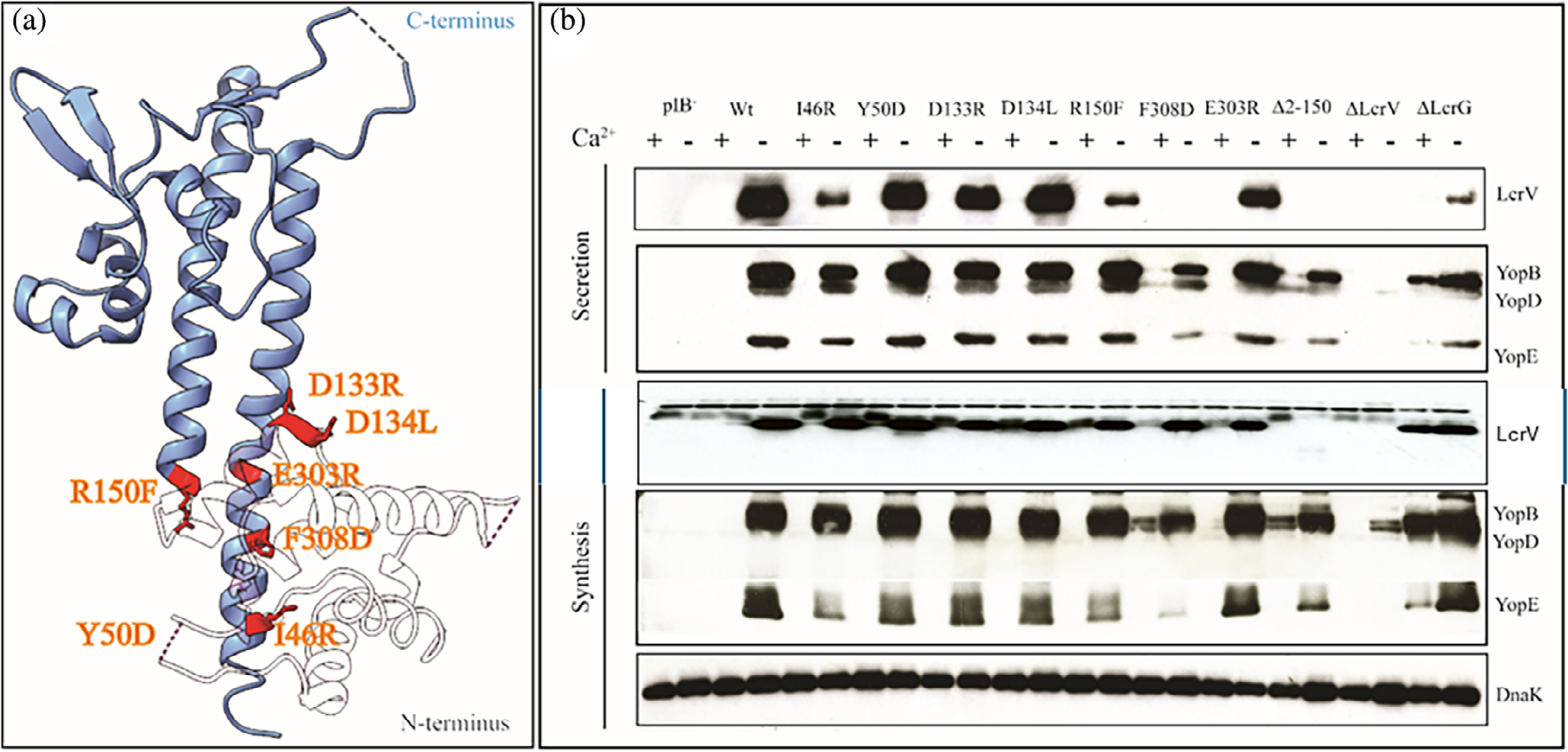
Phenotypic analysis of the T3SS in *Y. pseudotuberculosis*. (a) Representation of mutations selected in this study is highlighted on the LcrV crystal structure (PDB ID: 1R6F; Derewenda et al., 2004). (b) In vitro secretion and synthesis profiles of the *lcrV* mutants on T3SS. Bacteria were grown in BHI media in the presence and absence of calcium. Proteins in the bacterial pellet (total synthesis) and supernatant (secretion) fractions were run on a 12% SDS PAGE and immunoblotted using anti‐YopB, anti‐YopD, anti‐YopE, anti‐LcrV and anti‐DnaK antisera. Strains are designated as follows: PIB^−^ YPIII plasmid cured; Wt, Wild type YPIII/pIB102; I46R, LcrV I46R YPIII/pIB1905; Y50D, LcrV Y50D YPIII/pIB1906; D133R, LcrV D133R YPIII/pIB1907; D134L, LcrV D134L YPIII/pIB1908; R150F, LcrV R150F YPIII/pIB1909; E303R, LcrV E303R YPIII/pIB1924; F308D, LcrV F308D YPIII/pIB1925; Δ2‐150, LcrV Δ2‐150 YPIII/pIB1901; ΔLcrV, LcrV Δ10‐313 YPIII/pIB19; ΔLcrG, LcrG Δ26‐66 YPIII/pIB701; Experiments were repeated at least thrice (*n* = 3).

In light of the proposed conformational change of the LcrV N‐terminus, we probed its functional relevance by quantifying the phenotypes of key LcrV variants with a standard in vitro Ysc‐Yop synthesis and secretion assay (Bergman et al., 1991; Francis & Wolf‐Watz, 1998). Cultivation of *Y. pseudotuberculosis* at 26°C and in the presence of 2.5 mM CaCl_2_ results in minimal synthesis or secretion of LcrV and Yops (Bergman et al., 1991). In contrast, the temperature upshift to 37°C together with simultaneous chelation of calcium induces the synthesis and secretion of LcrV and Yops (Bergman et al., 1991). Whole bacteria and cell‐free supernatant fractions from the growth‐standardized cultures were assessed using immunolabeling with monospecific antibodies in a western blot assay (Costa et al., 2010; Francis & Wolf‐Watz, 1998). This in vitro effect has been suggested to mimic the contact between *Yersinia* and eukaryotic host cells that triggers a polarized transfer of effector proteins (Pettersson et al., 1996). Typically, a null‐mutant in LcrV renders a phenotype with severely attenuated synthesis and secretion of effector proteins (Pettersson et al., 1999). Here we have reproduced these results as a reference for the new LcrV variants studied herein (Figure 6b). We started by analyzing mutant bacteria expressing the LcrV_Δ2–150_ variant lacking the entire N‐terminal domain. This mutant was severely attenuated for secretion of LcrV, and decreased synthesis of Yops (exemplified by YopB, YopD, and YopE). The reduction in synthesis indicated that the regulatory role assigned to LcrV is compromised (Skrzypek & Straley, 1995).

We examined seven additional substitutions in the N‐terminus (I46R, Y50D, D133R, D134L, and R150F) and in the coiled‐coil domain (E303R and F308D) (Figure 6a) for their secretion phenotype in *Y. pseudotuberculosis*. These mutants were selected based on the interaction interface of the LcrV‐LcrG complex as shown by the HDX‐MS experiments. LcrV mutants (I46R and R150F) showed a slight decrease in the secretion of the tested Yops as well as LcrV (Figure 6b) while the synthesis remains on par with that of the wild type. Benchmarking against the LcrV_Δ2–150_ variant, both I46R and R150F retain the low secretion phenotype as observed for LcrV_Δ2–150_, while the synthesis is significantly higher in I46R and R150F. These results suggest that the low secretion and low synthesis phenotype of the LcrV_Δ2–150_ variant to some degree can be decoupled with the I46R and R150F replacements. However, at this point we cannot suggest a molecular mechanism that enables this decoupling. Similarly, mutations in the coiled‐coil domain of LcrV (F308D) also appeared to affect the secretion of LcrV and the other Yops tested (Figure 6b). This shows that mutations in the N‐terminus and C‐terminus of LcrV affect distinct outcomes, either on substrate secretion or regulatory control on T3SS substrate synthesis and secretion. Although the mutations were predominantly based on our finding that the N‐terminus of LcrV is dynamic, the biological result does not necessarily reflect the effects of the LcrV/G complex itself but may be reflecting properties of LcrV in the assembled T3SS injectosome, At this point we can postulate that the N‐terminus of LcrV is linked to function and that this segment undergoes a dynamic change upon complex formation with LcrG, but a linkage between these effects is currently not established.

### 2.6 Yeast two‐hybrid protein–protein interaction analysis of LcrV and LcrG

**FIGURE 7:**
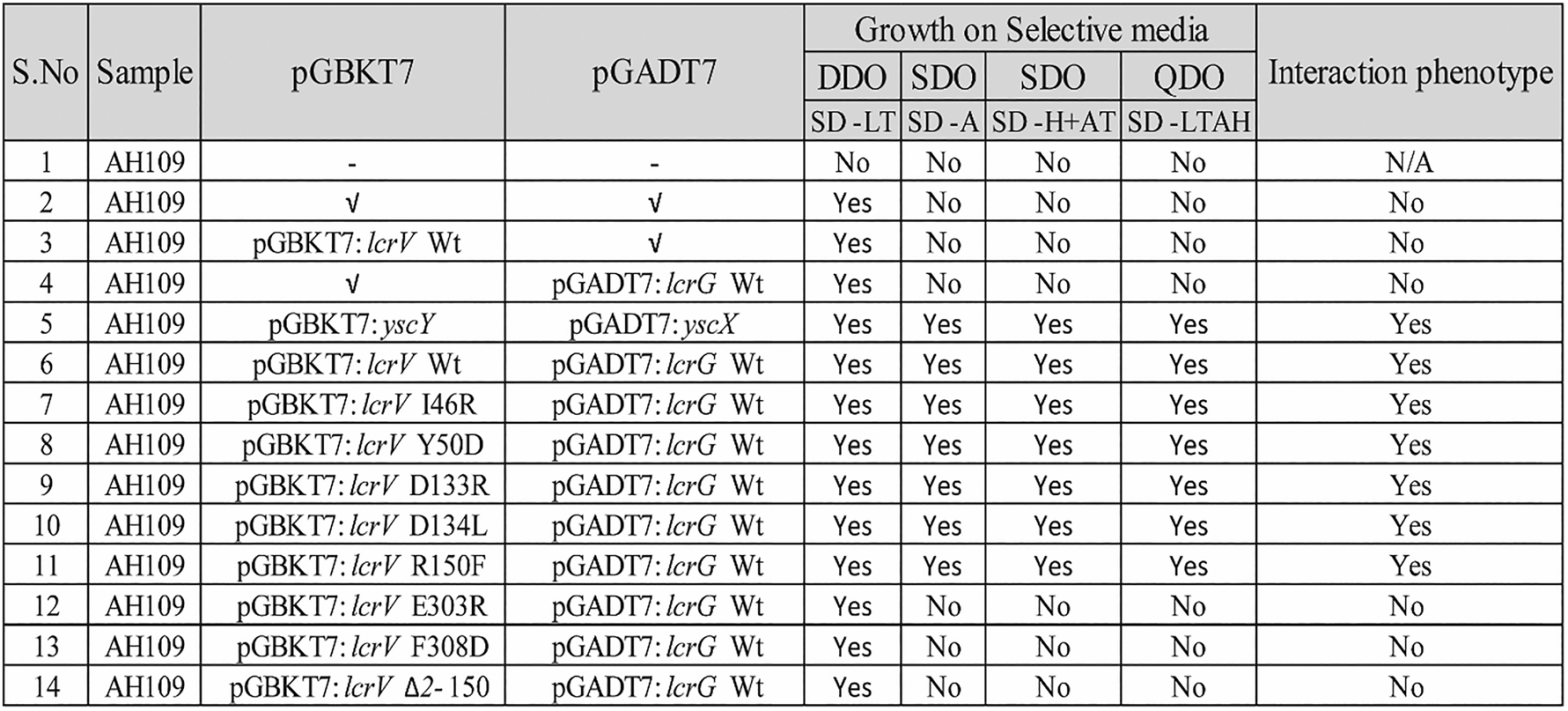
Summary of yeast two‐hybrid protein interaction experiments. Analysis of *lcrV* (wild type or mutants) with *lcrG* wild type. *S. cerevisiae* (AH019) was transformed with pGBKT7 and pGADT7 (empty vectors or fused to desired LcrV, LcrG or LcrV mutants) and selected on synthetic dropout media: −Leucine, −Tryptophan, (SD^−LT^). Interaction of the LcrG and LcrV is assessed through their growth on the synthetic dropout media: −Adenine (SD^−A^); −Histidine + 1 mM AT (SD^−H+AT^); and −Leucine, −Tryptophan, −Adenine and –Histidine (SD^−LTAH^). Experiments were performed at least thrice (*n* = 3).

Protein–protein interactions in vivo were examined using the yeast two‐hybrid system to assess whether the gene products of *lcrV* and *lcrG* mutations differ in their interaction profiles. Wild type and mutant constructs of *lcrG* and *lcrV* were cloned with the activation domain and binding domain in the pGADT7 and pGBKT7 vectors, respectively. Yeast was transformed with pGBKT7:*lcrV* wild type or mutants along with pGADT7:*lcrG* wild type, grown on selective media, and assayed for their interaction by measuring growth on the selective media. No interaction was observed in the yeast transformed with empty vector controls. In contrast, positive interactions were observed in yeast with the positive control YscX and YscY, as well as with LcrV and LcrG (Figures 7 and S5). Due to a lack of stable expression in yeast (Figure S6) we were unable to observe an interaction between LcrG and truncated LcrV_Δ2–150_ (Figures 7 and S5). Mutations in LcrV N‐terminus (I46R, Y50D, D133R, D134L, and R150F) did not affect the interaction with LcrG. However, substitution mutations in the C‐terminus (E303R and F308D) showed a decreased or disrupted interaction with the wild type LcrG (Figures 7 and S5), although in the case of F308D, this could be explained by a lack of stable product (Figure S6). This suggests that the coiled‐coil domains of LcrV may be involved in its interactions with LcrG in vivo.

## 3 DISCUSSION

Molecular insight into LcrV is warranted in light of the importance of LcrV in effector secretion (Hamad & Nilles, 2007; Ligtenberg et al., 2013; Sarker et al., 1998) and as a potential drug target (Biryukov et al., 2023; Cowan et al., 2005; Hill et al., 1997; Hotinger & May, 2020). By combining structural biology and biophysics methods with functional analysis, we provide evidence that LcrV is a dynamic protein that undergoes a conformational change in its N‐terminal domain upon complex formation with LcrG. Functional assays uncovered that a deletion of the N‐terminal domain resulted in a low secretion and low synthesis phenotype. By making specific substitutions (I46R and R150F), the low secretion and synthesis outcomes could, in part, be decoupled. Further studies are required to understand the structural mechanisms that enable this phenotypic decoupling. Overall, the results suggest that the N‐terminal domain has an important contribution to the regulation of the T3SS in *Y. pseudotuberculosis*. It should be noted, however, that these functional effects are unlikely to reflect intrinsic properties of the LcrV/G complex itself, but rather are more likely attributable to LcrV as it is incorporated within the assembled T3SS injectosome. HDX‐MS analysis of the LcrV–LcrG complex did not reveal a well‐defined interaction surface on LcrV. This suggests that the interaction may be multivalent in nature and potentially involves regions of structural disorder. The high affinity between LcrV and LcrG with a *K*_D_ of 127 nM, suggests that multivalent interactions in protein–protein recognition can retain strong binding. An extreme example of this property is the ultra‐high affinity interaction between the intrinsically disordered proteins histone H1 and prothymosin‐α, where structural disorder is retained within their complex (Borgia et al., 2018).

A titration model for the regulation of the T3SS has been proposed where the repressive effect of LcrG is relaxed by formation of a complex with LcrV (Nilles et al., 1998), and where surplus LcrV is directed to the injectisome tip. A notable feature of the LcrV–LcrG interaction is that the N‐terminal domain of LcrV changes conformation to support formation of the complex. Based on FRET data and the observation that the N‐terminal domain adopts a folded conformation in isolation, the conformational change appears to involve a rigid‐body rotation around a hinge located at the domain's terminus (Figure 3). Unexpectedly, ^19^F NMR analysis of the LcrV–LcrG interaction revealed that LcrV samples minor structural states in both its free and LcrG‐bound forms. The chemical shifts of these states suggest that unbound LcrV may transiently adopt a conformation resembling the LcrG‐bound structure, and conversely, that LcrG‐bound LcrV may retain features of the unbound form (Figure 4). This behavior aligns with the conformational selection or population shift model, where an unbound state explores structural ensembles like those of the binding‐competent state (Gianni et al., 2014; Kovermann et al., 2017). This is an expansion of the classic induced fit model where the interactions themselves drive conformational change (Koshland, 1958). The detection of minor conformational states in LcrV indicates that it is a dynamic protein, and that these dynamics likely contribute to the formation of the LcrV–LcrG complex.

After discovering that the dynamic nature of the N‐terminal domain is an inherent characteristic of LcrV, we focused on analyzing protein–protein interactions and the functional impact of specific amino acid substitutions in this domain. None of the N‐proximal variants tested in vivo influenced the interaction with LcrG. However, mutations in the C‐terminal region (residues Glu303 and Phe308) seemed to affect the interaction with LcrG, at least as measured by the yeast two‐hybrid system. These results are consistent with previous studies indicating that mutations in the coiled‐coil domain of LcrV disrupt its interaction with LcrG (Hamad & Nilles, 2007; Lawton et al., 2002). Furthermore, we observed that the secretion of LcrV is reduced in the LcrV Glu303 and Phe308 mutants. Notably, the distinct phenotype associated with the LcrG deletion mutant, characterized by loss of calcium‐dependent regulation and constitutive Yop expression under non‐permissive conditions, was not observed in the LcrV mutants that lack interaction with LcrG. This suggests that additional regulatory factors may contribute to the phenotypic differences between LcrV and LcrG mutants, warranting further investigation.

## 4 MATERIALS AND METHODS

### 4.1 Strains, plasmids, and growth conditions

Bacterial strains and plasmids used in this study are listed in Tables S1 and S2, respectively. Culturing of bacteria was performed routinely in Lysogeny broth (LB) or LB agar, either at 26°C for *Y. pseudotuberculosis* or at 37°C for *Escherichia coli* with aeration (180 rpm). Whenever required, bacterial culture media were supplemented with the following antibiotics: Carbenicillin (100 μg/mL), chloramphenicol (25 μg/mL), and kanamycin (50 μg/mL). For yeast two‐hybrid assays, *Saccharomyces cerevisiae* (AH109) was maintained in YEPD broth or agar (2% peptone, 1% yeast extract, 2% glucose) at 30°C.

### 4.2 Generation of LcrV and LcrG constructs for protein expression

The *lcrG* (wild type), *lcrV* (wild type, *lcrV*Δ_1–150_, *lcrV*Δ_1–150+300–326_, *lcrV*Δ_300–326_) genes were sub‐cloned into either the pNIC28‐Bsa4 or pET24d expression vectors. These modified plasmids with the desired *lcrV* or *lcrG* gene constructs contain a His tag with an integrated TEV cleavage site and a resistance gene for Kanamycin. These plasmids were further transformed into *E. coli* BL21 (DE3) expression strain. *E. coli* cells carrying the desired *lcrG* or *lcrV* (wild type or variants) were all grown individually in 10 mL LB media supplemented with kanamycin at 37°C (180 rpm). Overnight cultures were sub‐cultured into 1 L fresh LB media and upon reaching an optical density (OD_600_) ~0.5–0.6, bacterial cultures were shifted to 18°C and expression of the proteins was induced with the addition of Isopropyl β‐D‐Thiogalactopyranoside (IPTG) at a final concentration of 0.42 mM. After overnight expression, the cells were harvested at 5000 rpm for 15 min at 4°C (Beckman Coulter, Avanti J‐26S) and the bacterial pellets were resuspended in either lysis buffer (50 mM Tris, 300 mM NaCl, 10 mM Imidazole, 1 mM TCEP, pH 7.4) or PBS (with 2 mM DTT, pH 7.4) to keep the proteins reduced.

Bacterial cells were lysed using sonication (Branson digital sonicator) for 30 s (8 times) with a 15 s break, and the cell debris was removed by ultracentrifugation at 45,000 rpm for 45 min at 4°C (Beckman Coulter, OPTIMA™). The resulting supernatant was applied onto a 5 mL His‐trap column (Cytvia) and eluted with a gradient of 30–500 mM imidazole. Furthermore, the excess imidazole was removed using dialysis membranes in dialysis buffer (20 mM Tris, 200 mM NaCl, 1 mM TCEP, pH 7.4). The dialyzed protein sample was further concentrated using Amicon® Ultra centrifugal filters (Merck) with a cut‐off of 3 and 10 kDa for LcrG and LcrV, respectively. Upon determination of the concentration of protein in the Nanovue (DeNovix) spectrophotometer the samples were either used for experiments directly or were flash frozen in liquid nitrogen for long term storage at −80°C.

### 4.3 Isothermal titration calorimetry

ITC experiments were performed using a MicroCal Auto‐iTC200 isothermal titration calorimeter (GE Healthcare). The reference cell was filled with the sample buffer (30 mM phosphate buffer, 50 mM NaCl, 1 mM TCEP, pH 7.0) and 200 μL of LcrG (21 μM) was added into the sample cell and titrated with 40 μL of LcrV (210 μM) loaded in the syringe. All the experiments were performed at 25°C, with the following test parameters: an initial injection volume of 0.5 μL was followed by 2 μL of titrant (LcrV) added for 19 injections to the sample cell, with a wait time of 300 s between the injections. As a quality control measure, an initial run was performed with 0.4 mM ethylenediaminetetraacetic acid (EDTA) and 5 mM CaCl_2_. Additionally, all the experimental runs included a pre‐water, post‐water and sample (LcrG or LcrV) with the buffer control only. The experiments were repeated at least three times.

### 4.4 Production of ^19^F enriched protein samples

For production of ^19^F‐labeled LcrV samples, *E. coli* BL21 (DE3) cells transformed with the full‐length *lcrV* construct were grown at 37°C in M9 minimal medium until an optical density (OD_600_) of around 0.5 was reached. Protein expression was induced with 0.4 mM IPTG simultaneously as 5‐fluoroindole (60 mg/mL) was added to the cultures (Crowley et al., 2012; Verma et al., 2022). The induced cells were grown at 18°C overnight and the following protein isolation and purification was performed as described earlier.

### 4.5 ^19^F NMR spectroscopy

The samples consisted of 500 μM ^19^F‐labeled LcrV in a buffer containing 20 mM Tris (pH 7.4), 200 mM NaCl, 1 mM TCEP and 20% D_2_O (v/v). For the LcrV‐LcrG complex, unlabeled LcrG prepared in the same buffer but without D_2_O was added in a 1:2 molar ratio. All experiments were performed at 25°C on a 600 MHz Bruker Avance III HD spectrometer (with a ^19^F Larmor frequency of 564 MHz) equipped with a 5 mm ^19^F BBO cryoprobe. The one‐dimensional (1D) spectra for both the free LcrV and LcrG bound form were acquired with the π/2 pulse length set to 14.7 μs, an acquisition time of 36 ms, a relaxation delay of 4 s and 4096 scans.

### 4.6 FRET analysis

Fluorescence steady‐state data were collected by using a Jasco FP‐6500 spectrofluorometer. All samples had an LcrV concentration of 1.5 μM either with or without attached BODIPY. In samples containing the LcrV‐LcrG complex, LcrG was present at 15 μM. All data were recorded in a 50 mM Tris–HCl buffer at pH 7.5. The temperature was kept constant at 25°C.

The donor, Trp, was excited at 280 nm (bandwidth = 3 nm) and its fluorescence emission spectra were recorded between 310 and 370 nm. The maximum fluorescence was detected at 339 nm and hence the value for this wavelength was used in the distance calculations. The fluorescence spectrum of the acceptor BODIPY was recorded between 480 and 540 nm with a maximum at 511 nm. Also, in this case the maximum value was used for the distance calculations. To avoid fluorescence reabsorption the absorbance of the Trp and BODIPY samples was less than 0.1. Moreover, the influence of photobleaching was found negligible on the experimental timescale.

### 4.7 Hydrogen‐deuterium exchange mass spectrometry

HDX protein–protein interaction analysis was performed on LcrV–LcrG at a molar ratio of 1:1 in TBS (50 mM Tris–HCl, 150 mM NaCl, pH 7.5). The HDX‐MS runs were performed for both LcrV apo state and LcrG apo state, and LcrV–LcrG interaction states in triplicate samples. A detailed methodology is provided in Data S1 and the summary of HDX experimental detail is reported in Table S4.

### 4.8 HDX‐MS data analysis

HDX data analysis and visualization were initially performed using HDExaminer, version 3.1.1 (Sierra Analytics Inc., Modesto, USA). Peptides identified by PEAKS with a peptide score value of −log_10_ (P) >25 and no modifications were used to generate a peptide list containing peptide sequence, charge state, and retention time for the HDX analysis. A detailed analysis is provided in Data S1. The obtained data were further analyzed in Deuteros 2.0 (Lau et al., 2021) to obtain significant peptides (*p* < 0.01).

### 4.9 Size exclusion chromatography coupled to multi‐angle light scattering

LcrV full length and LcrV truncated (LcrV_Δ1–150_) were analyzed by SEC‐MALS using an ÄKTA pure (Cytiva) coupled to a miniDAWN TREOS II detector and OptiLab T‐rex online refractive index detector (Wyatt Technology). The absolute molecular mass was calculated using the light scattering data with the ASTRA analysis software package, version 7.2.2.10 (Wyatt Technology). BSA was used for calibration and the proteins were separated on a Superdex 200 increase 10/300 GL analytical SEC column (Cytiva) with a flow rate of 0.4 mL/min. For both variants, 200 μL were injected at a concentration of 10 mg/mL in 50 mM NaCl, 30 mM phosphate buffer and 1 mM TCEP. The refractive index increment was set at 0.185 mL/g and the extinction coefficient for UV detection at 280 nm was calculated from the primary structure of the proteins: 0.4997 (mL/mg.cm) and 0.4575 (mL/mg.cm), respectively for LcrV full length and LcrV truncated.

### 4.10 Generation of lcrV in cis mutants in *Y. pseudotuberculosis*

Amino acid substitution and deletion mutants in *Y. pseudotuberculosis* were obtained using the methods described earlier (Francis et al., 2017). Briefly, PCR was performed with the primer pairs listed in Table S3, to generate either point or deletion mutations in *lcrV*. An overlap PCR was used to amplify the *lcrV* DNA fragments flanked by SpeI and SacI‐HF restriction enzyme sites and was cloned into the pGEM®T vector (Promega). The generated DNA constructs were confirmed by sequencing (Eurofins MWG Operon). Sequence‐verified constructs were subcloned into the pDM4 vector. These mutagenesis vectors were further transformed into *E. coli* S17‐1λ*pir* and subsequently used for conjugal mating with *Y. pseudotuberculosis* and allowed for primary and secondary allelic exchange events (Francis et al., 2017). Finally, all the mutants were verified and confirmed by a PCR amplification of the *lcr*V sequence encoding the mutation, and subsequently by sequencing analysis of the PCR product.

### 4.11 Yeast two‐hybrid assay

Both wild type *lcr*V, *lcr*G and mutant gene constructs of *lcr*V were PCR amplified using primers mentioned in Table S3. PCR amplified constructs were then cloned into the pGEM®T sequencing vector and sequenced verified as mentioned earlier. Sequence confirmed constructs of *lcr*V and *lcr*G (wild type or mutants) were then sub‐cloned into EcoRI‐BamHI RE sites of the yeast two‐hybrid vectors, where *lcr*V and *lcr*G gene constructs are fused to the GAL4 DNA binding domain in pGBKT7 and GAL4 activation domain in pGADT7 (Clonetech), respectively.

To assess the interactions, yeast two‐hybrid vectors carrying the *lcr*V and *lcr*G alleles were transformed into the reporter strain *S. cerevisiae* AH109 as described elsewhere (Francis et al., 2000) and grown on double drop‐out synthetic media lacking tryptophan and leucine for 3–4 days at 30°C. Furthermore, 1–2 colonies for each transformed sample were resuspended in 100 μL sterile milliQ water and subjected to five‐fold serial dilutions. A 5 μL suspension of sample from each dilution was transferred onto four sets of plates: (a) Double drop‐out (DDO‐SM‐Leu‐Trp); (b) Triple dropout media (TDO‐SM‐Leu‐Trp‐Ade); (c) Triple dropout media (TDO‐SM‐Leu‐Trp‐His + 4 mM 3‐aminotriazole (3‐AT)); (d) Quadruple dropout media (TDO‐SM‐Leu‐Trp‐Ade‐His + 4 mM 3‐AT); and were allowed to grow for 3–4 days at 30°C. Protein–protein interactions were determined by measuring the growth of transformed yeast with the desired plasmid constructs on triple drop‐out synthetic media devoid of tryptophan and leucine as well as adenine or histidine supplemented with 4 mM 3‐AT. Empty vectors alone, pGADT7/*lcrV*:pGBKT7, and pGBKT7/*lcrG*:pGADT7, and untransformed *S. cerevisiae* AH109 were used as negative controls, whereas known interacting pairs of *yscY*:pGBKT7 and *yscX*:pGADT7 were used as the positive control (Bröms et al., 2005). The described Y2H assay used for studying the LcrV and LcrG interactions was repeated at least three independent times.

### 4.12 Synthesis, secretion, and detection of the type III‐secreted substrates in *Y. pseudotuberculosis*

Analysis of the T3SS substrate synthesis and secretion in *Y. pseudotuberculosis* was performed as described earlier (Francis & Wolf‐Watz, 1998). *Yersinia* were grown overnight in Brain Heart Infusion (BHI) in the presence and absence of Ca^2+^ at 26°C. The bacterial cultures were standardized based on the growth (OD_600_) and subcultured in their respective fresh media for 1 h at 26°C. Subsequently, the cultures were shifted to 37°C for 3 h for maximal expression and secretion of the T3SS substrates. The cultures were normalized based on the OD_600_, and the protein content associated with the whole bacteria and cell‐free supernatant was obtained. Total bacterial protein and cell‐free extracts were further subjected to SDS‐PAGE (12% acrylamide), transferred onto PVDF membranes (Immobilon®, Merk Millipore) and detected with primary rabbit monoclonal or polyclonal antibodies against YopD, YopB, YopE, and LcrV (AgriSera AB). For chemiluminescent detection, anti‐rabbit secondary antibodies conjugated with Horse Radish Peroxidase (GE Healthcare), followed by ECL2 western blotting substrate detection kit (Thermo scientific) were used.

## AUTHOR CONTRIBUTIONS

**Jagadish Chandra Kumar Mangu:** Investigation; methodology; formal analysis; visualization; writing – original draft; writing – review and editing. **Per Rogne:** Investigation; writing – original draft; writing – review and editing; visualization; methodology; formal analysis. **Jonna Mattsson:** Methodology; investigation; formal analysis; visualization; writing – original draft; writing – review and editing. **Lucas Hultgren:** Investigation; formal analysis; writing – original draft; visualization; writing – review and editing; methodology. **Kumar D. Gahlot:** Visualization; formal analysis; methodology; investigation; writing – review and editing. **Anaïs Lamy:** Investigation; visualization; formal analysis. **Ronnie P.‐A. Berntsson:** Visualization; investigation; writing – original draft; writing – review and editing; formal analysis. **Lennart B.‐Å. Johansson:** Methodology; formal analysis; investigation; visualization; writing – original draft; writing – review and editing. **Matthew S. Francis:** Conceptualization; supervision; funding acquisition; project administration; resources; writing – original draft; writing – review and editing. **Magnus Wolf‐Watz:** Conceptualization; supervision; funding acquisition; project administration; resources; writing – original draft; writing – review and editing.

## CONFLICT OF INTEREST STATEMENT

The authors declare no conflicts of interest.

## Supporting information


**Figure S1.** Illustration of the activation and regulation of the type three secretion system. (Top) Under environmental conditions or at lower temperatures (e.g., 25°C), the regulatory protein YmoA suppresses T3SS gene expression by inhibiting the master transcriptional activator LcrF (Chen et al., 2016; Schwiesow et al., 2016). Within the bacterial cytoplasm, LcrG is believed to inactivate LcrV by forming a high‐affinity complex (Matson & Nilles, 2001). Upon entry into a mammalian host or exposure to elevated temperatures (e.g., 37°C), the early needle complex begins to assemble (Dewoody et al., 2013), illustrated here with components YopN and TyeA. During this stage, LcrG is hypothesized to inhibit premature T3SS activation from within the cytoplasm. The assembly of late‐stage components, including the needle filament protein YscF and the tip complex proteins YopB, YopD, and LcrV, is triggered when extracellular calcium is no longer sensed. At this point, LcrV is proposed to sequester LcrG away from the secretion machinery, thereby facilitating full activation of the T3SS. Once the tip complex is inserted into the host cell membrane, Yop effector proteins, along with tip complex components, are translocated into the host cytoplasm, where they contribute to bacterial virulence and immune evasion. (Bottom) Table summarizing the universal nomenclature and specific protein names for the needle tip, translocon, and needle filament components. Created in BioRender by Mangu, J. (2025).
**Figure S2.** Isothermal titration calorimetry analysis on the interaction of the Δ1‐23 LcrV with full length LcrG. Experiments were performed at least thrice (*n* = 3).
**Figure S3.** Overview of the peptide coverage in the HDX‐MS data and the significant peptides in LcrV and LcrG: (A, C) Coverage of the peptides across LcrV and LcrG in apo and complex states. (B, D) Significant and non‐significant peptides in LcrV and LcrG (*p* < 0.01) which are deprotected or protected across four‐time scales (a = 30s; b = 300 s; c = 3000 s; d = 6000 s).
**Figure S3.** Trimeric LcrV_Δ1–150_ engages in a 1:1 complex with LcrG. Analytical gel filtration of the complex (black) formed by mixing trimeric free LcrV_Δ1–150_ (red) and monomeric LcrG (blue), demonstrates that the complex migrates as a significantly smaller particle compared to trimeric LcrV_Δ1–150_.
**Figure S5.** Yeast two‐hybrid protein interaction analysis of LcrV (wild type or mutants) with LcrG wild type. *lcrV* and *lcrG* are cloned into pGBKT and pGADT7 vectors respectively and are transformed into *S. cerevisiae* AH109. Protein interaction between LcrG and LcrV are assessed through their growth on the synthetic dropout media (SD‐Ade and SD‐His +1 mM AT). Experiments were performed at least thrice (*n* = 3).
**Figure S6.** Expression of LcrV and LcrG hybrids in *Saccharomyces cerevisiae* AH109. Protein extracts were generated from yeast alone (AH109), harboring the empty Y2H expression plasmids (pGBKT7 and pGADT7) or plasmids containing *lcrV* allelic variants encoding LcrV wild type and mutated versions (pGBKT7::LcrV_WT_, pGBKT7::LcrV_I46R_, pGBKT7::LcrV_Δ2–150_, pGBKT7::LcrV_Y50D_, pGBKT7::LcrV_D133R_, pGBKT7::LcrV_D134L_, pGBKT7::LcrV_R150F_, pGBKT7::LcrV_E303R_, or pGBKT7::LcrV_F308D_) and *lcrG* allele encoding LcrG wild type (pGADT7::LcrG_WT_). The *lcrV* alleles were fused to the GAL4 DNA binding domain plasmid pGBKT7. The *lcrG* allele was fused to GAL4 activation domain of pGADT7. Samples were separated by SDS‐PAGE, and recombinant proteins were identified by immunoblot analysis using a rabbit polyclonal antibody raised against purified LcrV (α‐LcrV panel) or LcrG (α‐LcrG panel) or a mouse monoclonal antibody from Thermofisher Scientific targeting PGK1 (α‐PGK1 panel). The latter was used as a sample loading control. The arrowheads indicates the detection of the protein bands of interest.
**Table S1.** Strains used in the study.
**Table S2.** Plasmids used in the study.
**Table S3.** Oligonucleotides used in the study.
**Table S4.** Overview of the peptide map overlap in the respective state.
**Data S1.** Raw HDX‐MS data set of LcrG apo and LcrG:LcrV (1:1).
**Data S2.** Raw HDX‐MS data set of LcrV apo and LcrV:LcrG (1:1).

## Data Availability

The data that supports the findings of this study are available in the supplementary material of this article.
