# Supplementary material for: Dynamic interaction of the Yersinia pseudotuberculosis type three secretion system proteins LcrV and LcrG
Source: Protein Sci. 2025 Dec 22;35(1):e70400. doi: 10.1002/pro.70400 (PMC12720783; doi:10.1002/pro.70400)
Supplement: Supplementary file 1 — Figure S1. Illustration of the activation and regulation of the type three secretion system. (Top) Under environmental conditions or at lower temperatures (e.g., 25°C), the regulatory protein YmoA suppresses T3SS gene expression by inhibiting the master transcriptional activator LcrF (Chen et al., 2016; Schwiesow et al., 2016). Within the bacterial cytoplasm, LcrG is believed to inactivate LcrV by forming a high‐affinity complex (Matson & Nilles, 2001). Upon entry into a mammalian host or exposure to elevated temperatures (e.g., 37°C), the early needle complex begins to assemble (Dewoody et al., 2013), illustrated here with components YopN and TyeA. During this stage, LcrG is hypothesized to inhibit premature T3SS activation from within the cytoplasm. The assembly of late‐stage components, including the needle filament protein YscF and the tip complex proteins YopB, YopD, and LcrV, is triggered when extracellular calcium is no longer sensed. At this point, LcrV is proposed to sequester LcrG away from the secretion machinery, thereby facilitating full activation of the T3SS. Once the tip complex is inserted into the host cell membrane, Yop effector proteins, along with tip complex components, are translocated into the host cytoplasm, where they contribute to bacterial virulence and immune evasion. (Bottom) Table summarizing the universal nomenclature and specific protein names for the needle tip, translocon, and needle filament components. Created in BioRender by Mangu, J. (2025). Figure S2. Isothermal titration calorimetry analysis on the interaction of the Δ1‐23 LcrV with full length LcrG. Experiments were performed at least thrice (n = 3). Figure S3. Overview of the peptide coverage in the HDX‐MS data and the significant peptides in LcrV and LcrG: (A, C) Coverage of the peptides across LcrV and LcrG in apo and complex states. (B, D) Significant and non‐significant peptides in LcrV and LcrG (p < 0.01) which are deprotected or protected across four‐time scales (a = [file PRO-35-e70400-s001.docx]

# **Supplementary Information**

## **Supplementary methods**

### **FRET Sample preparation**

The plasmid for the D293C/C273A double mutant of LcrV (pNIC-Bsa4 LcrV(C273A/D293C)) was obtained from the protein expertise platform (PEP) at Umeå University. The plasmid was transformed into BL21(DE3) and protein expressed. The protein was purified in a reducing environment (2 mM DTT) to keep the cysteine reduced. BODIPY™ FL Iodoacetamide (Sigma Aldrich) was dissolved in DMSO to a final concentration of 12 mM. LcrV, with the DTT removed, was mixed with BODIPY at a 10 times excess of BODIPY and the reaction was incubated at room-temperature over-night. The excess of BODIPY was removed using a PD-10 column. The labelling efficiency was determined by comparing the absorbance of the protein at 280 nm and the BODIPY at 503 nm using spectrophotometry.

### **FRET analysis**

##### **Theoretical considerations**

The analysis of steady-state fluorescence data assumes the classical Förster Resonance Electronic Transfer (FRET) theory of electronic energy (Förster, 1949) transport between one donor (Trp, denoted W) and one acceptor (BODIPY, hereafter denoted B). The W and B groups are mutually separated at a distance $R_{V}$ within the LcrV protein molecule. W-B electronic energy transfer was observed as the steady-state ratio between the W fluorescence intensity in the presence and absence of B, which is given by

$\frac{F_{\mathrm{WB}}(\lambda_{\mathrm{Wfl}})}{F_{W}(\lambda_{\mathrm{Wfl}})}=\frac{1}{1+k_{T}\tau_{W}}$ (1a)

In eq. 1 $k_{T}$ , $\tau_{W}$ stands for the rate of energy transfer and fluorescence lifetime of W, respectively. The transfer rate

$k_{T}=\frac{3}{2\tau_{W}}\left\langle\kappa^{2} \right\rangle\left[ \frac{R_{0}}{R} \right]^{6}$ (1b)

The average angular orientation $\left\langle\kappa^{2} \right\rangle$ is taken to be the isotropic value of 2/3, and the Förster radius was calculated for this value, whereby the Förster radius *R*_0_=34.6 ± 0.3 Å (Olofsson et al., 2006).

In all experiments the degree of BODIPY-labelling, $\delta$ = 0.43. Overall, the fluorescence steady-state experiments intend to account for the transfer efficiency when comparing intensity ratios calculated in the presence and absence of LcrG. For the donor and acceptor and the fluorescence intensities are

$F_{\mathrm{WB}}\propto N_{W}\left[ 1-\delta+\frac{\delta}{1+{(\frac{R_{0}}{R})}^{6}} \right]$ (2a)

$F_{\mathrm{BW}}\propto N_{W}\frac{{(\frac{R_{0}}{R})}^{6}}{1+{(\frac{R_{0}}{R})}^{6}}$ (2b)

, respectively. $N_{W}$ and $N_{W}^{´}$ denote the steady-state number of excited Trp groups, in the absence $\left( N_{W} \right)$ and presence $\left( N_{W}^{´} \right)$ of LcrG, respectively. The ratio $\left( \frac{N_{W}}{N_{W}^{´}} \right)$was determined from the Trp fluorescence intensities monitored in absence of the BODIPY group.

##### **W-B distances in LcrV obtained in the presence and absence of LcrG complex**

The following different ratios between experimental emission steady-state intensities were analyzed namely, $\frac{F_{W}^{\exp}}{F_{\mathrm{WB}}^{\exp}}$, $\frac{F_{\mathrm{WG}}^{\exp}}{F_{\mathrm{WBG}}^{\exp}}$, $\frac{F_{\mathrm{WB}}^{\exp}}{F_{\mathrm{BW}}^{\exp}}$,$\frac{F_{\mathrm{WBG}}^{\exp}}{F_{\mathrm{BWG}}^{\exp}}$, and $\frac{F_{\mathrm{BW}}^{\exp}}{F_{\mathrm{BWG}}^{\exp}}$ and ,$\frac{F_{\mathrm{WBG}}^{\exp}}{F_{\mathrm{WBG}}^{\exp}}$. In the suffix notation the first letter (W or B) refers to the fluorescence intensity of the emitting component, whereas the second one is the donor or acceptor component (B or W). G indicates the LcrG-LcrV complex. From the data of $\frac{F_{W}^{\exp}}{F_{\mathrm{WB}}^{\exp}}$ and $\frac{F_{\mathrm{WG}}^{\exp}}{F_{\mathrm{WBG}}^{\exp}}$ratios, the obtained W-B distances are *R*_V_ = 26.8 Å in the LcrV molecule, and *R*_VG_ = 26.5 Å in its complex with LcrG. Corresponding distances calculated from $\frac{F_{\mathrm{WB}}^{\exp}}{F_{\mathrm{BW}}^{\exp}}$ and $\frac{F_{\mathrm{WBG}}^{\exp}}{F_{\mathrm{BWG}}^{\exp}}$ are 28.8 Å and 29.2 Å, respectively. A combination of the ratios of $\frac{F_{\mathrm{BW}}^{\exp}}{F_{\mathrm{BWG}}^{\exp}}$ and $\frac{F_{\mathrm{WBG}}^{\exp}}{F_{\mathrm{WBG}}^{\exp}}$ give $R_{V}$ = 29.8 Å and *R*_VG_ = 33.0 Å from a combination of second order equations derived from.

Taken together, upon the formation of the LcrV-LcrG complex, FRET data are compatible with a minor increase in the overall distance between W-B in the complex. Notice, a distance is interpreted as that between the center of mass of the Trp and that of the BODIPY group.

### **Hydrogen-deuterium exchange mass spectrometry (HDX-MS)**

The HDX-MS analysis was made using automated sample preparation on a LEAP H/D-X PAL™ platform (Trajan Scientific and medical) interfaced to an LC-MS system, comprising an Ultimate 3000 micro-LC coupled to an Orbitrap Q Exactive Plus MS (Thermo Scientific). A 3 µL of HDX samples were diluted with 27 µL TBS or HDX labeling buffer of the same composition prepared in D_2_O (pH 7.1). The HDX labeling was carried out for t = 0s, 30s, 300s, 3000s, and 9000s at 4°C. The labeling reaction was quenched by dilution of 28 µL labeled sample with 28 µl of 1% TFA, 0.4 M TCEP, 4 M urea, pH 2.5 at 1°C. And 55 µL of the quenched sample was directly injected and subjected to online pepsin digestion at 4°C (in-house immobilized pepsin column, 2.1 x 30 mm). The online digestion and trapping were performed for 4 minutes using a flow of 50 µL/min 0.1 % formic acid, pH 2.5. The peptides generated by pepsin digestion were subjected to on-line SPE on a PepMap300 C18 trap column (1 mm x 15 mm) and was washed with 0.1% FA for 60s. Thereafter, the trap column was switched in-line with a C18 reversed-phase analytical column (Hypersil GOLD, particle size 1.9 µm, 1 x 50 mm) and separation was performed at 1°C using a gradient of 5-50 % B over 8 minutes, and then from 50 to 90% B for 5 minutes, the mobile phases were 0.1 % formic acid (A) and 95 % acetonitrile/0.1 % formic acid (B). Following the separation, the trap and column were equilibrated at 5% organic content, until the next injection. The needle port and sample loop were cleaned three times after each injection with mobile phase 5% MeOH/0.1% FA, followed by 90% MeOH/0.1% FA and a final wash of 5% MeOH/0.1% FA. After each sample and blank injection, the Pepsin column was washed by injecting 90 µl of pepsin wash solution 1% FA /4 M urea /5% MeOH. In order to minimize carry-over a full blank was run between each sample injection. Separated peptides were analyzed on a Q Exactive Plus MS, equipped with a HESI source operated at a capillary temperature of 250 °C with sheath gas 12, Aux gas 2, and sweep gas 1. For HDX analysis MS full scan spectra were acquired at 70K resolution, AGC 3e6, Max IT 200 ms, and scan range 300-2000. For identification of generated peptides separate undeuterated samples were analyzed using data-dependent MS/MS with HCD fragmentation.

### **HDX-MS data analysis**

PEAKS Studio X Bioinformatics Solutions Inc. (BSI, Waterloo, Canada) was used for peptide identification after pepsin digestion of undeuterated samples. The search was done on a FASTA file with sequences of both proteins, the search criteria were a mass error tolerance of 15 ppm and a fragment mass error tolerance of 0.05 Da, allowing for fully unspecific cleavage by pepsin. The data from LcrV-LcrG interaction run were analyzed and compared to the apo state of LcrV and with the apo state of LcrG. The analysis was made on charge states 1-6 for each peptide, allowed only for EX2, and the two first residues of a peptide were assumed unable to hold deuteration. Due to the comparative nature of the measurements, the deuterium incorporation levels for the peptic peptides were derived from the observed relative mass difference between the deuterated and non-deuterated peptides without back-exchange correction using a fully deuterated sample (Engen & Wales, 2015). As a full deuteration experiment was not made full deuteration was set to 75% of the maximum theoretical uptake. The presented deuteration data is the average of all high and medium confidence results. The allowed retention time window was ± 0.5 minutes. Heatmap settings were uncolored for proline, and heavy smoothing, and the difference heatmaps were drawn using automatically calculated significance based on replicate variance. The spectra for all time points were manually inspected; low-scoring peptides, obvious outliers, and any peptides with retention time correction that could not be made consistent were removed. As bottom-up labeling HDX-MS is limited in structural resolution by the degree of overlap of the peptides generated by pepsin digestion, the peptide map overlap is shown for the respective state in supplementary Table S4.

### **Analytical gel filtration**

For analytical gel filtration a Superose 12 10/300 GL column (Amersham)c was used. The column was equilibrated with sodium phosphate buffer (30 mM sodium phosphate, 50 mM NaCl, 1 mM TCEP, pH 7.0). Equal amounts of LcrV _Δ1-150,_ LcrG and LcrV _Δ1-150,_ LcrG 1:1 complex was loaded, and the column was eluted with 1 column volume of the equilibration buffer. The elution profile was monitored by following the absorbance at 280 nm.

**Alkaline lysis of Yeast for whole protein extraction and immunoblotting**

Yeast two-hybrid vectors carrying the *lcrV* and *lcrG* alleles were transformed into *S. cerevisiae* AH109 and selected on double drop-out synthetic media as described earlier in materials and methods. For analysis of the protein expression, from each transformant, 1-2 colonies were resuspended in 5 mL of synthetic media lacking tryptophan and leucine amino acids. At the same time, empty vector control (AH109) was grown in YEPD broth that contained yeast extract (1%), peptone (2%), and dextrose (2%). Yeast cultures (5 mL) were allowed to grow in 15 mL falcon tubes overnight at 30 °C with shaking at 150 rpm. Overnight cultures were normalized to an optical density (OD) of 600 nm (3.0 OD), and total protein content from each sample was extracted using the alkaline lysis method for Yeast with few modifications (MATSUO et al., 2006). Briefly, the normalized samples were centrifuged at 10,000 rpm for 2 min, and the harvested pellets were washed once with 1 mL of sterile water and resuspended in 200 µL of 0.1N NaOH. Resuspended pellets were incubated at room temperature (RT) for 20 min and centrifuged at 12,000 rpm for 5 min at 4 °C. Harvested pellets were mixed in 200 µL of SDS-PAGE sample buffer (Final 1X composition) and heat-denatured at 95 ^°^C for 8 min. Prepared samples were stored at -20 °C for subsequent immunoblot analysis.

Samples were thawed on ice, and 5 µL from each sample was loaded onto a precast SDS-PAGE Mini-PROTEAN® TGX™ gel (4-20%, Bio-Rad, Cat. #4561096) and run at 100 V at RT until the dye front reached the bottom of the gel. The gel was rinsed with double-distilled water and then submerged in 1× immunoblot wet transfer buffer (Trizma base, 3.02 g/L; glycine, 14.41 g/L; methanol, 5% v/v) for 10 min at RT. The entire gel was transferred to a polyvinylidene difluoride (PVDF) Immobilon®-P membrane (0.45 µm, Millipore) using the Bio-Rad Mini Trans-Blot® wet transfer system at 53 V for 1 h at 4 °C. Following transfer, the PVDF membrane was incubated in TBST buffer (Tris-buffered saline with 0.1% Tween-20) containing 5% (w/v) skimmed milk for 1 h at RT with gentle shaking to block non-specific proteins. After blocking non-specific proteins, the membrane was incubated overnight at 4 °C with gentle shaking in the primary antibody solution prepared in TBST containing 5% (w/v) skimmed milk. The primary antibody dilutions were prepared as follows: LcrV (1:10,000), LcrG (1:5,000), and PGK1 (1:5,000). The next day, the membrane was washed four to five times with TBST buffer (15 min each wash at RT with gentle shaking), then incubated for 1 h at RT with gentle shaking in the appropriate secondary antibody solution: ECL™ Anti-rabbit IgG-HRP (GE Healthcare; 1:5,000 dilution) for LcrV and LcrG, or ECL™ Anti-mouse IgG-HRP (Thermo Fisher Scientific; 1:5,000 dilution) for PGK1. After secondary incubation, the membrane was washed again four to five times with TBST buffer (15 min each at RT with gentle shaking). Finally, the membrane was incubated with 1 mL of Pierce™ ECL Plus Western Blotting Substrate (Thermo Fisher Scientific) for 5 min at RT and imaged using the LAS-4000 GelDoc system (Fujifilm) with automatic chemiluminescence settings.

## **Supplementary data**

### **Supplementary Figure S1:**


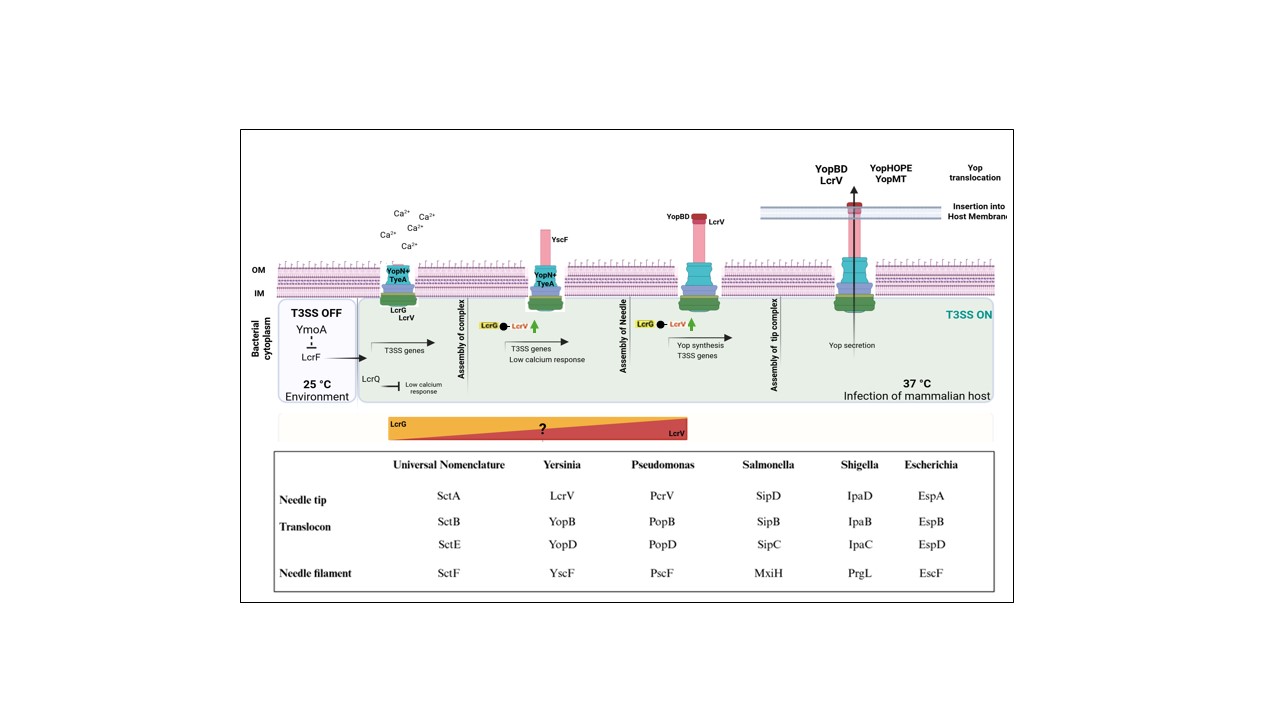


**Supplementary Figure S1.** Illustration of the activation and regulation of the type three secretion system. (Top) Under environmental conditions or at lower temperatures (e.g., 25 °C), the regulatory protein YmoA suppresses T3SS gene expression by inhibiting the master transcriptional activator LcrF (Chen et al., 2016; Schwiesow et al., 2016). Within the bacterial cytoplasm, LcrG is believed to inactivate LcrV by forming a high-affinity complex (Matson & Nilles, 2001). Upon entry into a mammalian host or exposure to elevated temperatures (e.g., 37 °C), the early needle complex begins to assemble (Dewoody et al., 2013), illustrated here with components YopN and TyeA. During this stage, LcrG is hypothesized to inhibit premature T3SS activation from within the cytoplasm. The assembly of late-stage components, including the needle filament protein YscF and the tip complex proteins YopB, YopD and LcrV, is triggered when extracellular calcium is no longer sensed. At this point, LcrV is proposed to sequester LcrG away from the secretion machinery, thereby facilitating full activation of the T3SS. Once the tip complex is inserted into the host cell membrane, Yop effector proteins, along with tip complex components, are translocated into the host cytoplasm, where they contribute to bacterial virulence and immune evasion. (Bottom) Table summarizing the universal nomenclature and specific protein names for the needle tip, translocon, and needle filament components. Created in BioRender by Mangu, J. (2025).

### **Supplementary Figure S2:**


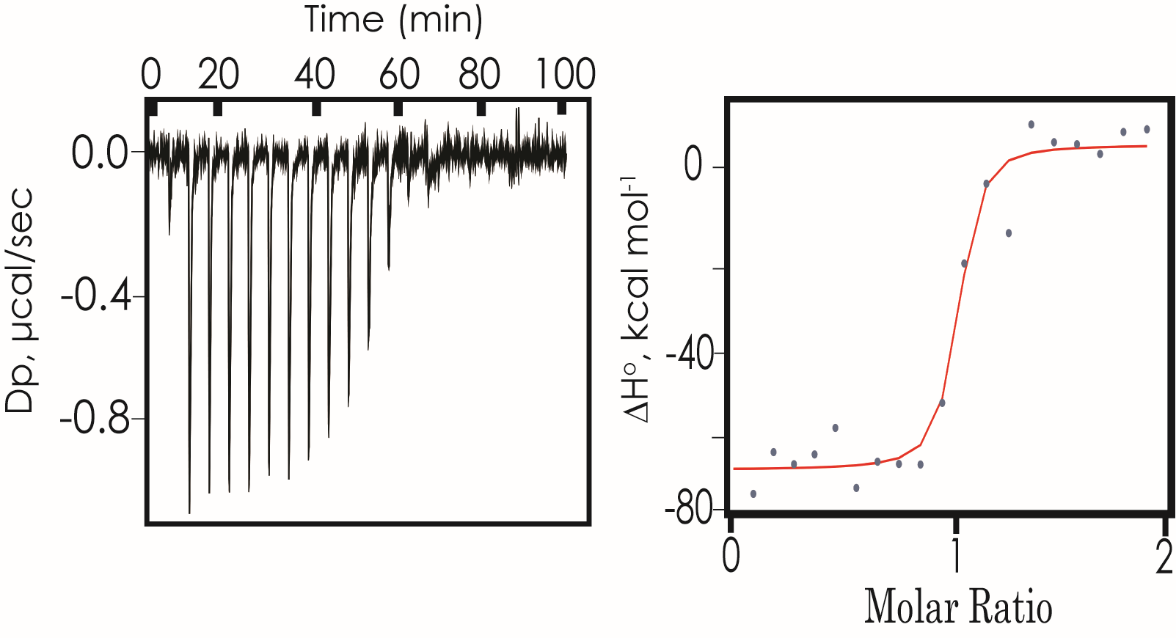


**Supplementary Figure S2**. Isothermal titration calorimetry (ITC) analysis on the interaction of the Δ1-23 LcrV with full length LcrG. Experiments were performed at least thrice (n=3).

### **Supplementary Figure S3:**


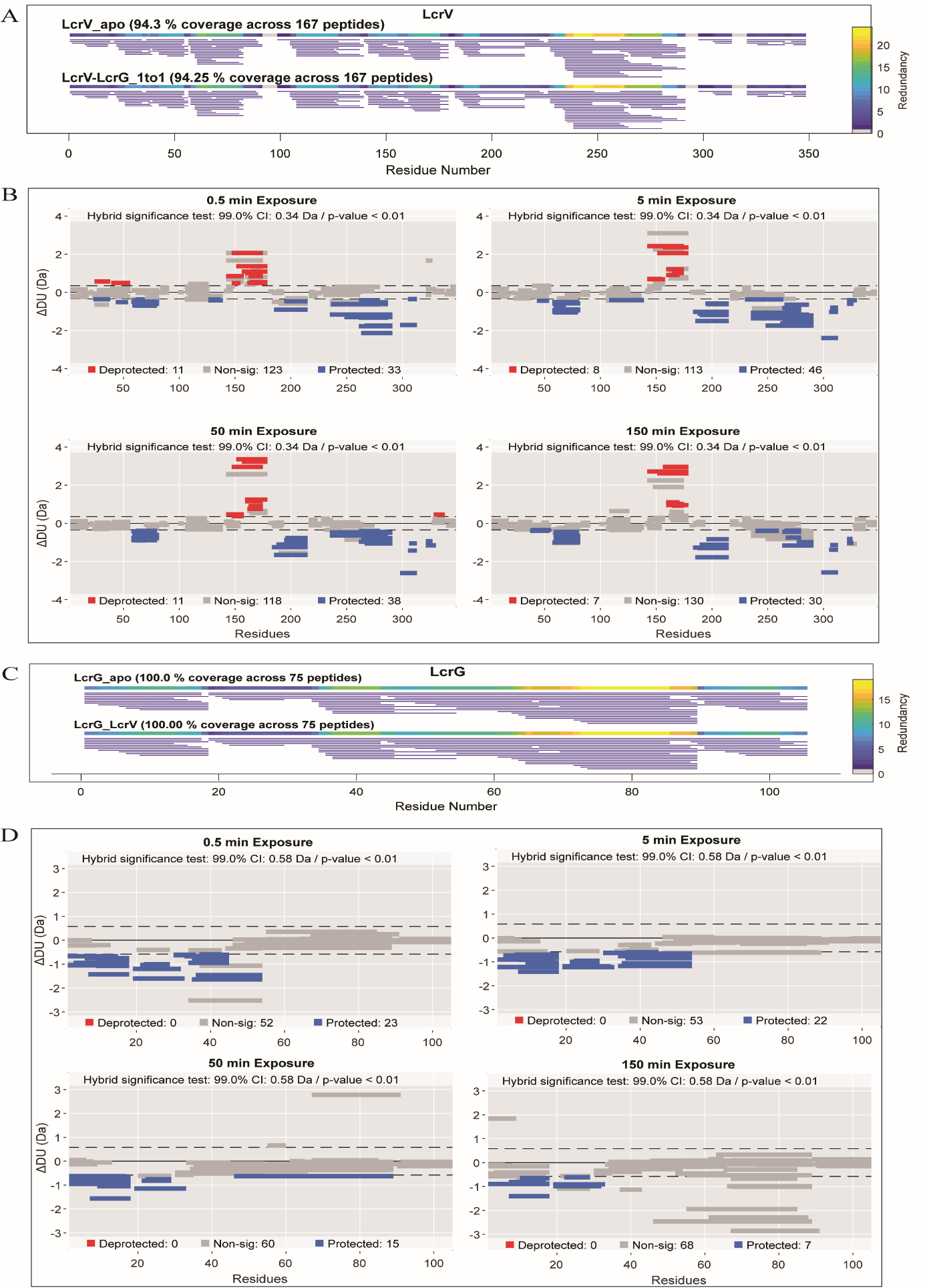


**Supplementary Figure S3**. Overview of the peptide coverage in the HDXMS data and the significant peptides in LcrV and LcrG: (**A, C**) Coverage of the peptides across LcrV and LcrG in apo and complex states. (**B, D**) Significant and non-significant peptides in LcrV and LcrG (p<0.01) which are deprotected or protected across four-time scales (a = 30s; b=300s; c=3000s; d=6000s).

### **Supplementary Figure S4:**


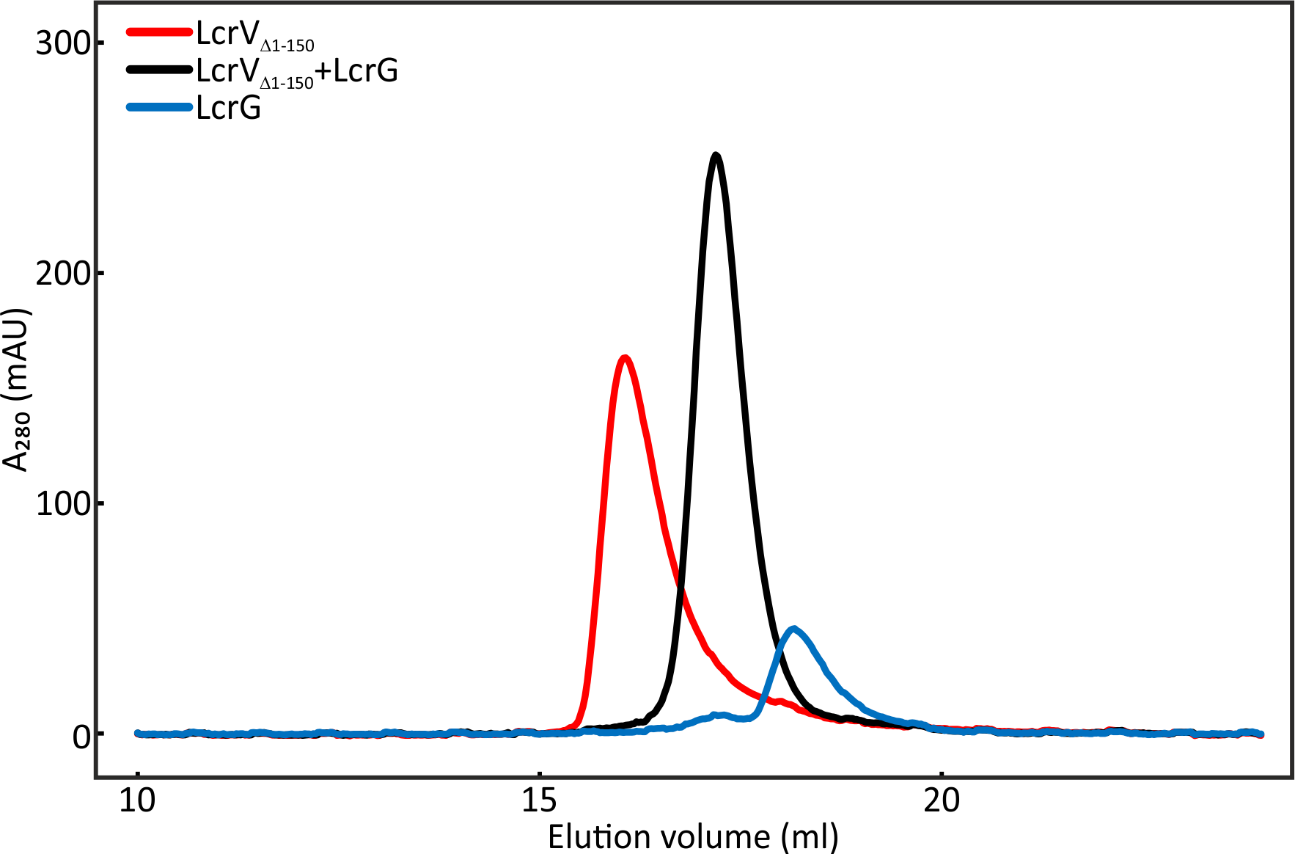


**Supplementary Figure S4. Trimeric LcrV_Δ1-150_ engages in a 1:1 complex with LcrG.** Analytical gel filtration of the complex (black) formed by mixing trimeric free LcrV_Δ1-150_ (red) and monomeric LcrG (blue), demonstrates that the complex migrates as a significantly smaller particle compared to trimeric LcrV_Δ1-150_.

### **Supplementary Figure S5:**


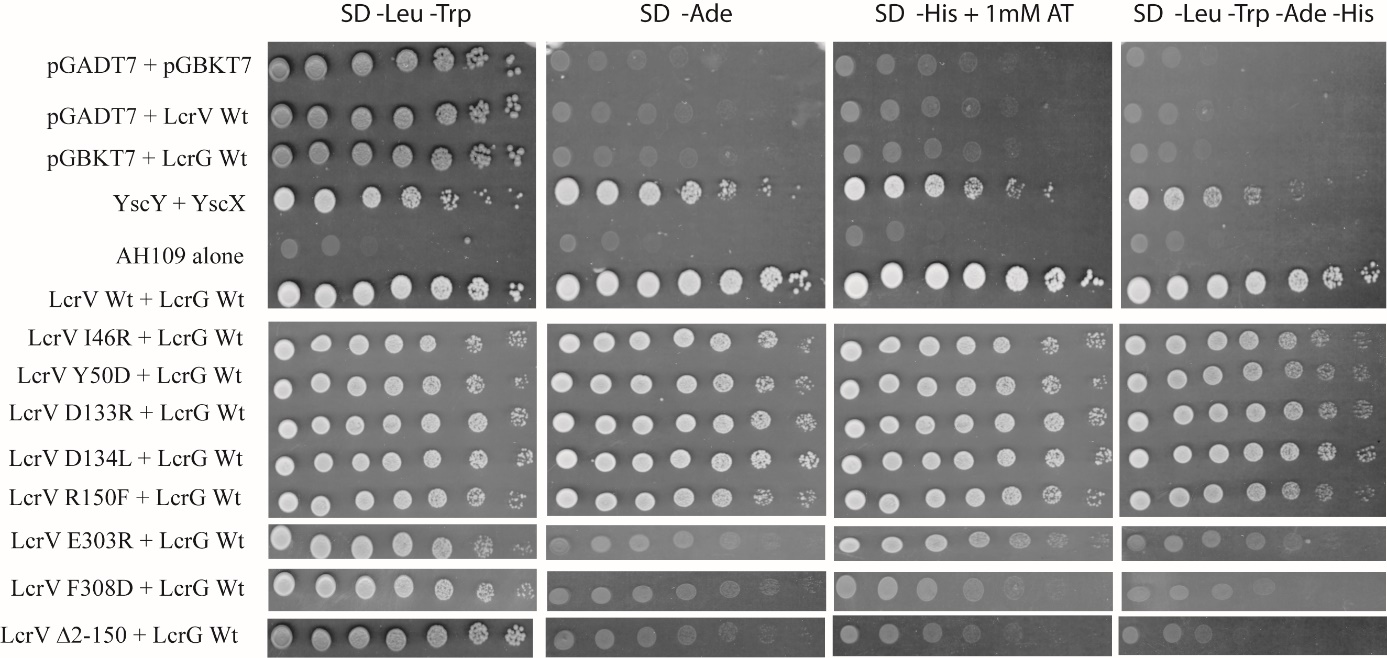


**Supplementary Figure S5**. Yeast two-hybrid protein interaction analysis of *lcrV* (wildtype or mutants) with *lcrG* wildtype. *lcrV* and *lcrG* are cloned into pGBKT and pGADT7 vectors respectively and are transformed into *S. cerevisiae* AH109. Interaction of the LcrG and LcrV are assessed through their growth on the synthetic dropout media (SD ^-Ade^ and SD ^-His + 1mM AT^). Experiments were performed at least thrice (n=3).


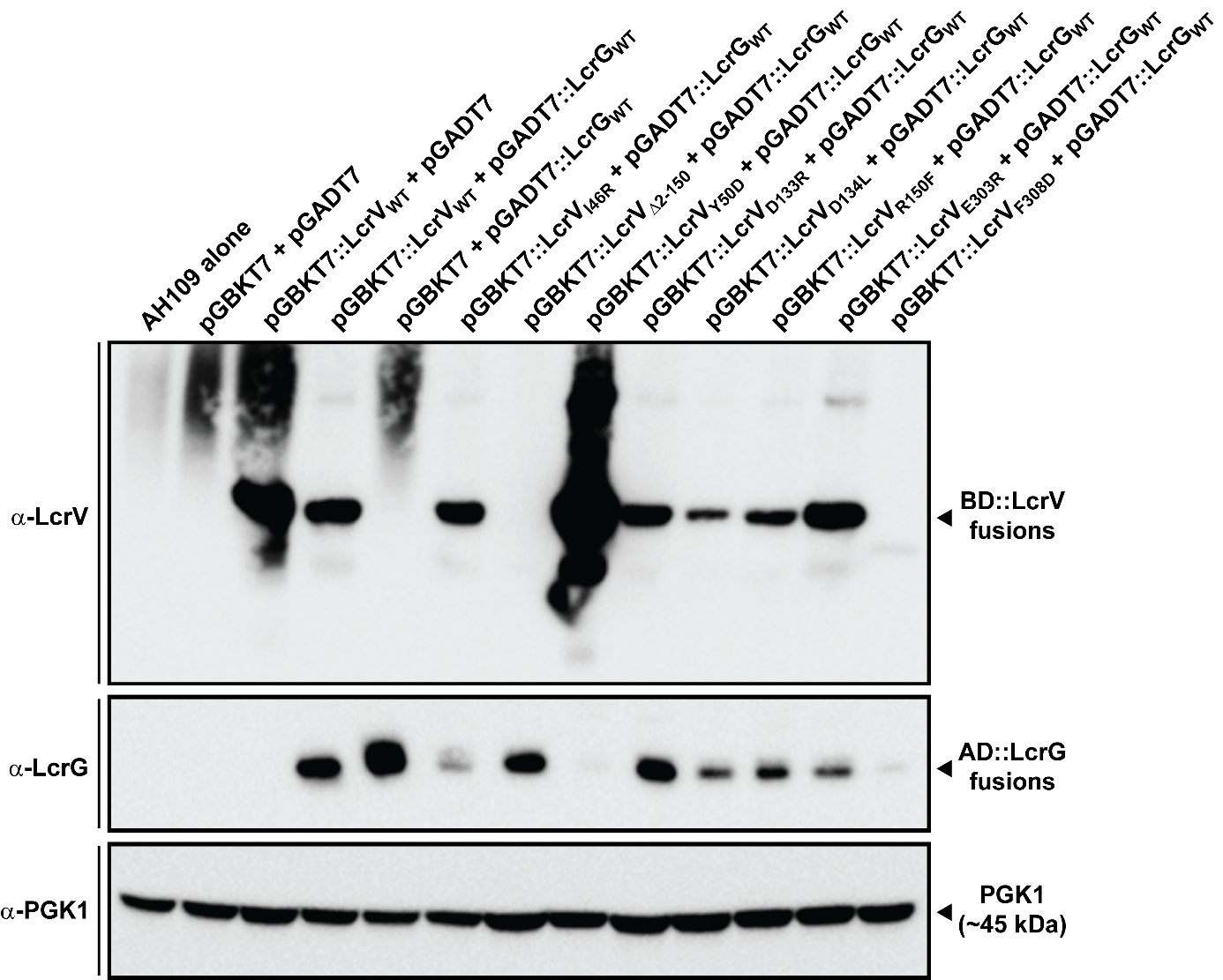


**Supplementary Figure S6.** Expression of LcrV and LcrG hybrids in *Saccharomyces cerevisiae* AH109. Protein extracts were generated from yeast alone (AH109), harboring the empty Y2H expression plasmids (pGBKT7 and pGADT7) or plasmids containing *lcrV allecic variants encoding* LcrV wild type and mutated versions (pGBKT7::LcrV_WT_, pGBKT7::LcrV_I46R_, pGBKT7::LcrV_Δ2-150_, pGBKT7::LcrV_Y50D_, pGBKT7::LcrV_D133R_, pGBKT7::LcrV_D134L_, pGBKT7::LcrV_R150F_, pGBKT7::LcrV_E303R_, or pGBKT7::LcrV_F308D_) and *lcrG* allele encoding LcrG wild type (pGADT7::LcrG_WT_). The *lcrV* alleles were fused to the GAL4 DNA binding domain plasmid pGBKT7. The *lcrG* allele was fused to GAL4 activation domain of pGADT7. Samples were separated by SDS-PAGE, and recombinant proteins were identified by immunoblot analysis using a rabbit polyclonal antibody raised against purified LcrV (α-LcrV panel) or LcrG (α-LcrG panel) or a mouse monoclonal antibody from Thermofisher Scientific targeting PGK1 (α-PGK1 panel). The latter was used as a sample loading control. The arrowheads indicates the detection of the protein bands of interest.

## **References**

Chen, S., Thompson, K. M., & Francis, M. S. (2016). Environmental Regulation of Yersinia Pathophysiology. *Front Cell Infect Microbiol*, *6*, 25. <https://doi.org/10.3389/fcimb.2016.00025>

Dewoody, R., Merritt, P. M., & Marketon, M. M. (2013). Regulation of the Yersinia type III secretion system: traffic control [Review]. *Frontiers in Cellular and Infection Microbiology*, *Volume 3 - 2013*. <https://doi.org/10.3389/fcimb.2013.00004>

Engen, J. R., & Wales, T. E. (2015). Analytical Aspects of Hydrogen Exchange Mass Spectrometry. *Annu Rev Anal Chem (Palo Alto Calif)*, *8*, 127-148. <https://doi.org/10.1146/annurev-anchem-062011-143113>

Förster, T. (1949). Experimentelle und theoretische Untersuchung des zwischenmolekularen Übergangs von Elektronenanregungsenergie. *4*(5), 321-327. <https://doi.org/doi:10.1515/zna-1949-0501> (Zeitschrift für Naturforschung A)

Matson, J. S., & Nilles, M. L. (2001). LcrG-LcrV interaction is required for control of Yops secretion in Yersinia pestis. *J Bacteriol*, *183*(17), 5082-5091. <https://doi.org/10.1128/jb.183.17.5082-5091.2001>

MATSUO, Y., ASAKAWA, K., TODA, T., & KATAYAMA, S. (2006). A Rapid Method for Protein Extraction from Fission Yeast. *Bioscience, Biotechnology, and Biochemistry*, *70*(8), 1992-1994. <https://doi.org/10.1271/bbb.60087>

Olofsson, M., Kalinin, S., Zdunek, J., Oliveberg, M., & Johansson, L. B. Å. (2006). Tryptophan–BODIPY: A versatile donor–acceptor pair for probing generic changes of intraprotein distances [10.1039/B601313A]. *Physical Chemistry Chemical Physics*, *8*(26), 3130-3140. <https://doi.org/10.1039/B601313A>

Schwiesow, L., Lam, H., Dersch, P., & Auerbuch, V. (2016). Yersinia Type III Secretion System Master Regulator LcrF. *Journal of Bacteriology*, *198*(4), 604-614. <https://doi.org/10.1128/jb.00686-15>
